# Protein Thermostability Prediction within Homologous Families Using Temperature-Dependent Statistical Potentials

**DOI:** 10.1371/journal.pone.0091659

**Published:** 2014-03-19

**Authors:** Fabrizio Pucci, Malik Dhanani, Yves Dehouck, Marianne Rooman

**Affiliations:** Department of BioModeling, BioInformatics and BioProcesses, Université Libre de Bruxelles, Brussels, Belgium; University of Michigan, United States of America

## Abstract

The ability to rationally modify targeted physical and biological features of a protein of interest holds promise in numerous academic and industrial applications and paves the way towards *de novo* protein design. In particular, bioprocesses that utilize the remarkable properties of enzymes would often benefit from mutants that remain active at temperatures that are either higher or lower than the physiological temperature, while maintaining the biological activity. Many *in silico* methods have been developed in recent years for predicting the thermodynamic stability of mutant proteins, but very few have focused on thermostability. To bridge this gap, we developed an algorithm for predicting the best descriptor of thermostability, namely the melting temperature 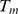, from the protein's sequence and structure. Our method is applicable when the 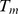 of proteins homologous to the target protein are known. It is based on the design of several temperature-dependent statistical potentials, derived from datasets consisting of either mesostable or thermostable proteins. Linear combinations of these potentials have been shown to yield an estimation of the protein folding free energies at low and high temperatures, and the difference of these energies, a prediction of the melting temperature. This particular construction, that distinguishes between the interactions that contribute more than others to the stability at high temperatures and those that are more stabilizing at low 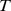, gives better performances compared to the standard approach based on 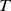-independent potentials which predict the thermal resistance from the thermodynamic stability. Our method has been tested on 45 proteins of known 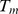 that belong to 11 homologous families. The standard deviation between experimental and predicted 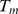
's is equal to 13.6°C in cross validation, and decreases to 8.3°C if the 6 worst predicted proteins are excluded. Possible extensions of our approach are discussed.

## Introduction

In the last decade there has been a growing attention on the study of the thermal stability of proteins and a lot of effort from both the theoretical and experimental sides have been devoted to understand its molecular basis. The potential applications are very broad and include the possibility to rationally modify the thermal stability of targeted proteins and hence optimize the bioprocesses in which they are involved [1]–[3]. This opens interesting perspectives in all academic and industrial sectors that exploit the unique properties of proteins, such as food industry, biofuel production, detergent industry, remediation of environmental pollutants, therapeutic approaches and drug design [4]–[6].

As a first step, it is quite important to gain theoretical understanding of the biophysical principles behind thermal stability. In a series of works [7]–[17] the mechanism and the interactions that promote or prevent thermal stabilization have been investigated. This is a highly non-trivial issue due to the large number of factors that influence the thermostability and to the marginal stabilization reached by the delicate balance between opposite energetic contributions. A series of factors has been indicated as responsible for the enhancement of the thermal resistance, based on the analysis of the amino acid conservation among the meso- and thermostable proteins belonging to the same homologous family. However, these factors are often not universal and family-dependent.

More general investigations of the factors that influence the thermal resistance have been performed using free energy calculations with a continuum solvation model [18]. They have led to the idea that salt bridges promote hyperthermostability in proteins, whereas they make little contribution to protein stability at room temperature. This idea is supported by a lattice model which suggested that salt bridges contribute not only on the stabilization of the native states but also to the destabilization of the misfolded conformations [19]. Moreover, on the basis of temperature-dependent statistical potentials, it has been shown that not only salt bridges, but also cation-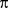 interactions, aromatic interactions, and hydrogen bonds between negatively charged and some aromatic residues tend to thermostabilize proteins, whereas hydrophobic packing appears to be neutral in this respect [20], [21].

Several approaches have been devised for designing mutants that are more thermally stable than wild-type proteins. Experimental methods include directed evolution, sometimes coupled with rational or semi-rational engineering strategies [22], [23]; for a review see [24] and references therein. *In silico* engineering approaches have also been developed, which are based on residue conservation within homologous families, on structural and dynamical features, or on free energy calculations [25]–[29]. A sequence-based *in silico* method for predicting melting temperatures has been developed and applied to distinguish hyperthermophilic from mesophilic microorganisms [30]. Even if these methods are partially successful, new, faster, more powerful and precise techniques would be welcome.

**Figure 1:**
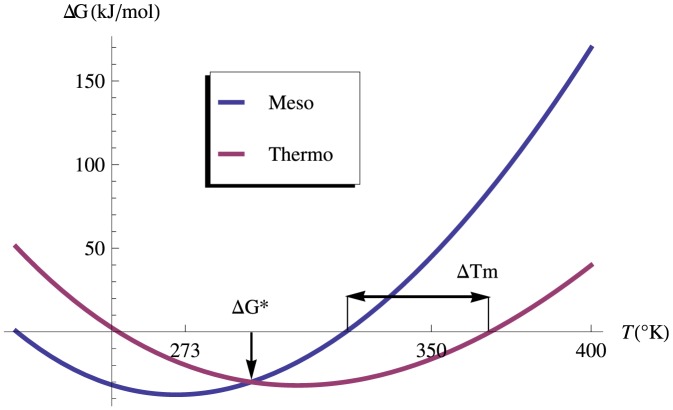
Thermal versus thermodynamic stability. An example of the stability curves of an hypothetical couple of mesostable and thermostable proteins, characterized by an equal thermodynamic stability at room temperature, but different thermal stabilities.

It is noteworthy that a lot more computational methods have been developed to predict the thermodynamic stability of a protein - in particular the thermodynamic stability changes upon point mutations (for review of their performances, see [31]–[34]). These are often used to also predict thermal stability, although thermal and thermodynamic stability are only very imperfectly correlated. Indeed, the thermodynamic stability at a given temperature is defined by the folding free energy 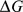 at that temperature, and the thermal stability by the melting temperature 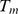. In Figure 1, one can find an example of the stability curves of two hypothetical proteins, one mesostable and the other thermostable, with approximately the same thermodynamic stability at room temperature (given by the 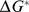 value) but with a significative difference in thermal stability (given by 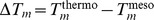) of about 50°C. There is thus a need to develop efficient and fast thermal stability predictors, without detour through thermodynamic stability.

**Figure 2:**
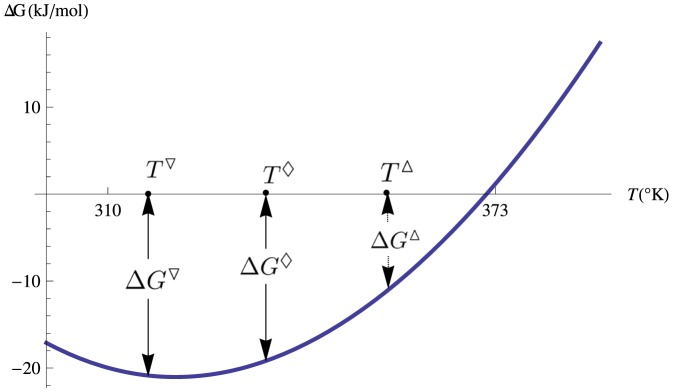
Folding free energies at different temperatures. Plot of the stability curve as a function of the temperature, and of the values of the three folding free energies 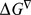, 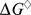 and 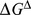 at the respective temperatures 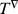, 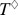, 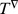, for a hypothetical protein.

The aim of this paper is to build an *in silico* method that directly predicts 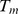, which is the best descriptor of thermal stability. For that purpose we have generalized and optimized the set-up introduced in [20], [21] for defining temperature-dependent statistical potentials. This set-up was originally devised for distance potentials that describe tertiary interactions, based on propensities of residue pairs to be separated by a certain spatial distance. Here we apply it to also define temperature-dependent torsion potentials, which describe local interactions along the polypeptide chain and are based on propensities of residues to be associated with backbone torsion angle domains [35]. The main idea behind the construction is that, since thermodynamic and thermal stability are not always correlated, some new potentials that are defined at different temperatures and thus take into account the thermal properties of the intra-protein interactions have to be introduced besides the standard statistical potentials that are defined at an average temperature. This construction is illustrated in Figure 2. The practical implementation consists of building different datasets of proteins with known melting temperature and deriving statistical potentials from each of these; because of the limited amount of data only two sets were considered, a mesostable and a thermostable one. Since there are not enough experimentally resolved structures with known 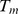, we have enlarged the datasets by introducing some proteins with unknown 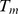 but for which a crude estimation of 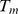 could be obtained from the environmental temperature of the host organism. This allowed us to derive smoother potentials and to obtain better performances.

**Figure 3:**
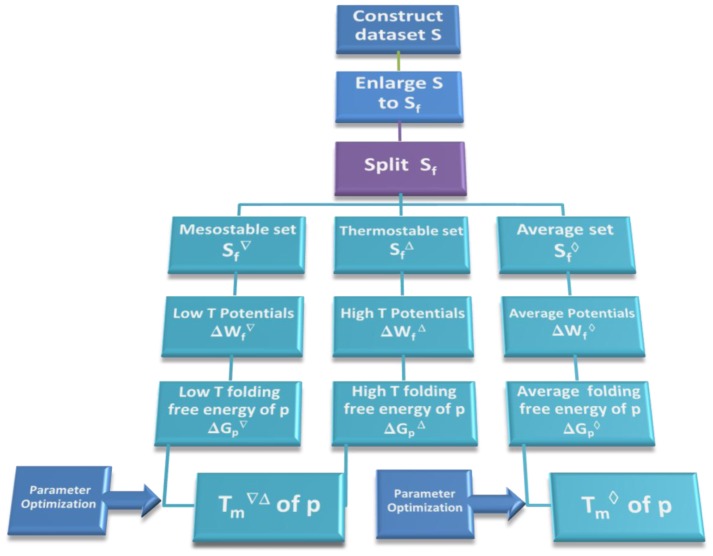
Flowchart of the 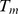 prediction method for a protein 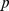 belonging to the family 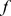.

Once the potentials were derived, they were used to give a quite accurate prediction of the melting temperature of a target protein, using additional information about the 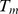 of homologous proteins. The overall flowchart of the method is summarized in Figure 3. Its performance was compared to that of the common procedure that uses temperature-independent potentials and hence predicts thermal resistance from thermodynamic stability.

## Methods

### Basic protein dataset 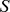 and homologous families

To define temperature-dependent potentials, we used the protein dataset defined in [20] and denoted as 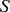, which contains 166 protein X-ray structures with resolution 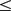
2.5 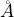 and known melting temperature 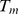 measured for the transition from the monomeric state to the denatured state. They were collected from the literature and the ProTherm database [36], and manually checked on the basis of the original articles. If several 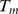-values were available for a given protein, we chose the 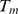 at the pH condition closest to 7; if different 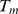
's were available at the same condition the average value was taken. In Table S0 in File S1 all the proteins belonging to this set and their characteristics are reported.

In this dataset, 11 families consisting of at least three homologous proteins were identified, whose melting temperatures will be predicted later in this paper and compared to the experimental melting temperatures. These are: 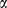-amylase, lysozyme, myoglobin, 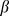-lactamase, 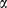-lactalbumin, acylphosphatase, adenylate kinase, cell 12A endoglucanase, cold shock protein, cytochrome P450 and ribonuclease.

### Enlarged, family-dependent, protein datasets 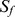


In view of constructing smoother potentials and designing a 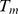-predictor that is specific for the proteins belonging to a given family 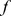, we have enlarged the basic dataset 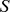. For each of the 11 families 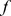, in turn, additional proteins belonging to 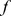 were added to the dataset 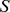 so as to create the family-dependent dataset denoted as 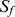. This procedure thus defines 11 different datasets 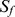, one for each family.

In contrast to the proteins from 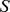, the 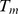
's of the additional proteins in 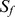 have not been characterized experimentally; only the environmental temperature of their host organism, 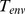, is known. This temperature refers to the optimal growth temperature for the micro- and cool-blooded organisms, while for the warm-blooded ones it is defined as the body temperature. The values of the 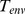 we are using (listed in Tables S1–S11 in File S1) were manually checked from the literature. When no optimal growth temperature was reported for a given microorganism, we took the mean of the range of temperatures over which it is able to grow.

In order to obtain an estimation of the melting temperature of these additional proteins, three different methodologies were used. We would like to stress that these estimations do not pretend to yield a reliable prediction of the 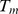, but they yield a rough approximation allowing us to decide if they belong to the set of thermostable or mesostable proteins, as explained later.

The first two methods for estimating the 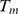
's are based on the environmental temperature 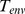. It is well known that 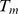 and 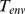 are correlated, since thermophilic organisms necessarily host thermostable proteins (even if the converse is not true). Based on experimental data on families of homologous proteins, a correlation between 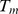 and 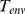 was indeed observed and the corresponding regression line was computed [38], [39]. The regression line obtained in [39] is:
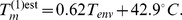
(1)The associated correlation coefficient, noted 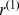 and computed without cross validation, is equal to 0.82. The 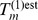
's derived with this formula are listed in Table S1–S11 in File S1.

However this correlation was derived regardless of the type of proteins. One can expect that inside a given family of homologous proteins the correlation between 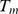 and 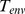 is stronger due to the fact that the thermostability is in some way related to specific protein characteristics. We thus calculated the linear regression between 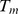 and 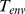 inside each family, even though the number of proteins per family is small and the statistical significance of the correlation questionable. The estimated 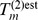
's so obtained are listed in Tables S1–S11 in File S1 and the regression lines for each family are given in Table S14 in File S1. The mean of the correlation coefficients 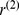 computed inside each family is equal to 0.84 (without cross validation) and is thus almost equivalent to the correlation coefficient 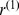 calculated on all families together. Note the peculiar case of the 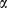-lactalbumin family (see Table S5 in File S1) for which the coefficients of the regression line are very different from the others. This family contains three proteins that belong to three warm-blooded organisms with very close 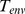
's (*Homo sapiens* 37°C, *Bos taurus* 38°C and *Capra hircus* 39°C) but 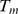
's that differ by more than 30°C. The 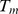-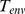 regression line obtained from these proteins is thus probably not reliable. The regression line of the lysosyme family is also atypical, but to a lesser extent.

The last method to estimate 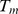
's is based on the sequence similarity between the proteins. We assign as 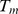 of a given protein the melting temperature of the protein of the same family that exhibits the highest sequence identity. This quite strong assumption is justified by the fact that, often, the higher the sequence identity, the higher the similarity among all structural, functional and thermodynamic characteristics, including thermostability. For that purpose, we performed pairwise alignments of all the sequences inside each family using the FASTA program [40]. The 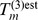
's estimated on the basis of these results are reported in Tables S1–S11 in File S1.

### Thermostable, mesostable and average protein datasets 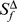, 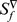 and 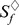


Each of the 11 family-dependent sets 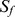 was divided into two equal subsets: the mesostable ensemble 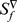 containing the proteins with (either known or estimated) 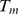 smaller than a certain threshold value 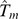 and a thermostable set 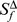 in which all proteins have 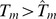. The threshold value 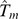 was determined in such a way that the two subsets contain an equal number of proteins; it thus slightly depends on 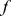.

Each subset was refined separately using the protein-culling server PISCES [37]. For each pair of proteins in a given subset that presents a sequence identity 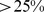, only one protein was kept according to the following criteria: (1) when one protein has a known 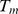 while the other has an estimated 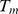 we chose the protein with known 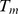; (2) when both proteins have either an experimentally determined 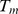 or an estimated 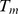, we chose the one with highest 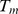 in the thermostable set and with lowest 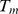 in the mesostable set. This procedure prevents significant sequence similarity to occur inside each subset, which could bias the predictions. It also allows us to increase the difference between the average melting temperatures 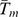 of the meso- and thermostable subsets, so as to get more differentiated temperature-dependent potentials.

We also constructed 11 family-dependent datasets 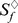 from 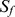. These sets were not split in two, but were refined using PISCES with the criterion that when two proteins (with both either known or estimated 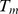) show a high degree of sequence identity (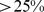), the protein with a melting temperature closest to the mean 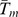 is kept and the other is discarded. This rule is not applied when one protein has an estimated 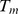 and the other a known 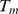; in such case the protein with known 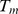 is kept and the protein with estimated 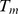 is discarded.

This procedure yields, for each of the 11 families 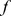, three protein datasets, a mesostable set 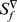, a thermostable set 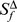, and an average set 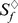. Each of these sets is characterized by 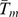, defined as the average of the melting temperatures of the proteins belonging to the set. This average temperature depends on the considered family. The dependence is, however, very small, and we will for the simplicity of the notations not add a subscript 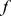 to 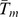. The values of the 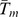
's associated to the different datasets are given in Table S13 in File S1.

### Stastistical potentials

Temperature- and family-dependent statistical potentials were derived from the datasets 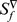, 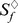, 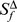, which are each characterized by a different average melting temperature 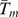. This is done using the Boltzmann law, following [20], [21]:
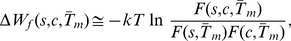
(2)where 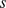 represent single amino acids or amino acid pairs, and 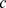 spatial distances between residue pairs or backbone torsion angle domains; 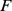 represent relative frequencies computed in the dataset of average melting temperature 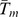, *i.e.* 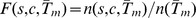.

In particular, we built two distance potentials and two torsion potentials. In the torsion potentials, 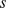 correspond either to the amino acid type 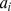 of residue 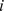 or to the amino acid types 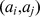 of residues 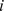 and 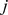, and 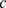 corresponds to the backbone torsion angle domain 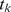 of residue 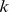. Seven 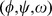 torsion angle domains were used, defined in [41]. These potentials describe local interactions along the chain: 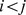 and 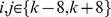. They are denoted as 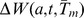 and 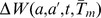.

In the two distance potentials, the structure motif 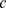 is the spatial distance 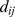 between the residues 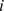 and 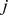, with 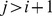. In 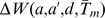, residues 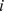 and 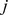 are of type 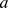 and 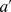. In 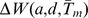, residue 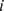 or 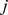 is of type 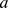 and the other is of arbitrary type. We defined the distance between two residues as the distance between the geometrical center of the heavy side-chain atoms [20]. The distance values between 3.0 and 8.0 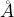 were grouped into 25 bins of 0.2 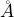 width; two additional bins describe distances larger than 8.0 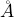 and smaller than 3.0 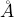, respectively. Moreover, we used a trick to artificially increase the number of occurrences in each bin and thereby smooth the potential. We summed the occurrences of neighboring bins, giving them a decreasing weight:
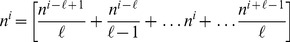
(3)where 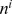 represents the number of occurrences 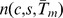 or 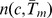 in bin 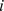, and 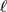 is set equal to 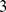; 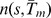 and 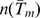 are normalized consequently.

In order to deal with the limited size of the datasets, a correction for sparse data [35] is applied:
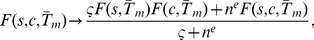
(4)where the expected number of occurrences is 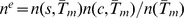, and 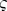 an adjustable parameter. This correction ensures that the potentials are close to 0 when the number of observations in the dataset is too small. The value of 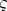 was chosen to be equal to either 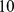 or 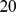.

We computed all the statistical torsion and distance potentials 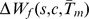 using the two values of 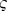 and the three different procedures for estimating 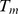 from 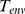, described in the previous subsections. This yields six different series of 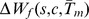
's. The final torsion and distance potentials that we consider in the following correspond to the average of these six potentials.

### Prediction of the melting temperature 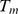


The folding free energy 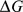 at some temperature referred to as 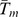 of a protein 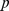 that belongs to the family 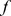 is evaluated by a linear combination of the four torsion and distance potentials defined in Eq. (2), which are derived from the sets of proteins (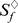, 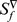 and 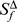) of average melting temperature 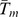:
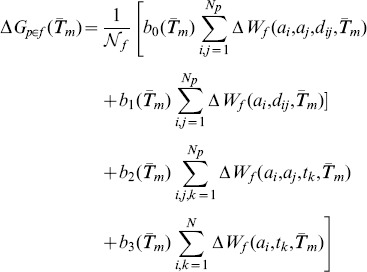
(5)where 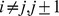 for the distance potentials, 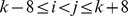 for the torsion potentials, 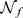 is a family dependent normalization factor, and 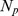 is the number of residues of 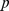. Let us for simplicity denote as 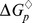, 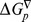 and 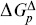 the family- and 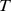-dependent folding free energies of protein 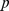 belonging to 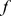 computed using the statistical potentiels derived from the sets 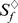, 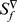 and 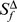, respectively.

We predict the melting temperature on the basis of these potentials in two different ways. In the first, we assume that the melting temperature is proportional to the average folding free energy 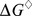. This is the common procedure that predicts thermal from thermodynamic stability. In the second, original, method, we assume that the melting temperature is proportional to the difference in folding free energy at two different temperatures: 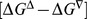. In these two procedures, the parameters, generically denoted as 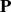, are optimized so as to minimize the standard deviation between the predicted and experimental melting temperatures of the ensemble of considered proteins; we use for that purpose the minimization function implemented in *Mathematica* 7. More precisely:
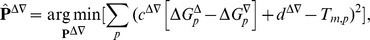


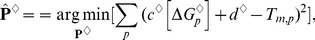
(6)where 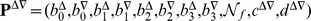 and 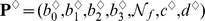; the sum over 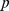 in these expressions means the sum over all the proteins with known melting temperature 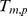 that belong to the 11 homologous families. The coefficients 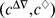 and 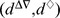 give, respectively, the slope and the intercepts of the regression line between computed folding free energies and experimental melting temperatures that best fit the data.

In order to avoid overestimating the performance of our method, we performed cross validation using the jack-knife technique: the parameters are identified on all proteins but one, which is used as test protein; every protein in turn is considered as test protein, and the average score is considered.

## Results

The contributions of amino acid interactions to protein stability are known to be temperature-dependent; some may be more stabilizing than others in the high temperature regime and less stabilizing than others at low 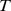, or conversely [18], [20], [21], [42], [43]. Such dependence need to be taken into account for a proper analysis of thermal stability properties. For that purpose, we created different datasets of proteins with known melting temperatures: in 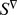 sets only mesostable proteins were considered, in 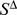 sets all entries are thermostable, and in 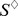 sets all proteins were taken independently of their 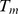. Each ensemble has been associated with a temperature 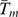 computed as the mean of the 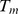 values of the proteins belonging to the set.

Predicting the melting temperature of a protein from its structure alone is quite a difficult task, and we therefore focus on the slightly simpler problem of predicting this temperature using information from homologous proteins. We hence selected 11 families of proteins of known 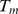, labelled by 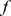, and defined 11 triplets of sets 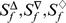, by adding proteins belonging to the family to the complete set 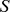, following the procedure explained in the Methods section.

From each of these datasets characterized by an average melting temperature 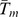, two torsion potentials and two distance potentials have been derived using the standard statistical-potential formalism that converts the relative amino acid frequencies into free energy trough the Boltzmann law (Eq.(2)). The torsion potentials are based on the propensities of single amino acids and amino acid pairs to adopt some backbone torsion angles and describe local interactions along the chain. The distance potentials describe tertiary interactions and are computed from propensities of amino acid pairs to be separated by a certain spatial distance. The total folding free energy 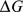 at some temperature 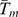 is explicitly computed as a linear combination of these different statistical potentials, derived from the dataset associated with 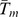 (Eq.(5)). We hence obtain, for each protein 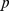, three folding free energies 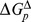, 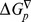 and 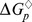; the coefficients of the combination are parameters that are fixed in a further step. In Figure 2 these three folding free energies at different temperatures 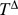, 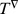 and 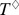 are depicted on the stability curve of a hypothetical protein.

Two procedures are used to predict the 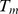
's from these free energies. The first assumes a linear correlation between 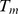 and 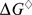, which is the standard way of predicting melting temperatures. The second, novel, procedure consists of assuming a linear correlation between 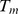 and 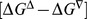. In the last step, the parameters (*i.e.* the coefficients of the linear combination of statistical potentials) were identified so as to minimize the difference between the computed and experimental 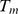
's (Eq.(6)). To avoid an overestimation of the performance, we systematically performed cross validations using the jack-knife technique as explained in the Methods section.

The first procedure, which assumes a correlation between 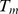 and 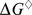, is justified by the fact that the thermodynamic and thermal stabilities are sometimes related, even if this is obviously not always true. Indeed, in the language of [44] (for a more recent review see also [45]), one way for the protein to enhance its thermostability is to increase its thermodynamic stability at all temperatures, thereby shifting the entire stability curve “downwards”, *i.e.* towards lower 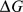
's. The other two ways to increase thermal resistance, namely a decrease of the heat capacity change 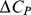 that brings a modification of the shape of the curve and a global shift of the curve towards the high temperature region, are instead better captured by the second procedure, which assumes a correlation between 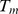 and the difference between the folding free energy at different temperatures, *i.e.* 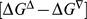.

**Figure 4:**
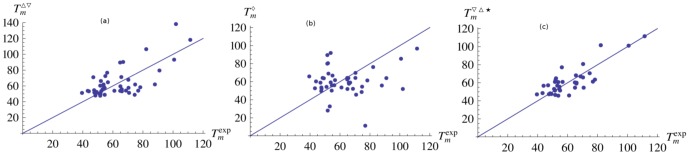
Melting temperature prediction. Relation between the experimental melting temperature 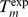 and the predicted temperatures: (a) 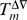 is computed from the folding free energy difference 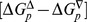 (correlation coefficient 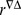 = 0.68), (b) 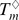 from the folding free energy 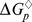 (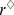 = 0.15), and (c) 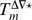 from 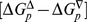 excluding the 6 proteins that are predicted worst (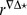 = 0.83).

The results of the 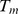 predictions for all proteins of our dataset are plotted in Figure 4. Figure 4.a shows the correlation between the experimental melting temperature and the temperature predicted from the folding free energy difference 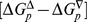. The associated linear correlation coefficient 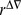 is equal to 0.68 (P-value 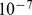). Figure 4.b shows instead the correlation between the experimental 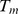
's and the 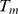
's predicted from the average potential 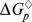. The corresponding linear correlation coefficient is very low: 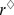 = 0.15 and is not statistically significant (P-value 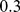). Clearly, the new procedure presented here, which predicts melting temperatures from 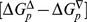 using 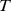-dependent statistical potentials, is much superior to the common procedure that predicts 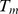 from 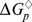 using simple 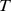-independent potentials.

Focusing on the 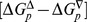-based method, we analyze whether some proteins are better predicted than others, and whether badly predicted proteins cause a significant decrease of the overall performance. In Figure 4.c, the 6 proteins that are predicted worst are excluded. To identify these proteins, we excluded at each step the protein whose melting temperature is predicted worst and we recompute the 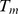
's of the remaining proteins. We repeat the procedure until 6 proteins are excluded. In this case the linear correlation coefficient rises up to 0.83 (P-value 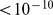).

The standard deviations 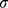 between the predicted and experimental values of the melting temperatures, computed for each family individually, are reported in Table 1; the results per protein are given in Table S12 in File S1. On average, 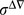 is equal to 13.6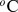 when computed on the basis of the free energy difference 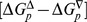. This is significantly better than the average 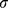-value computed with the standard 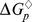-based method, which yields 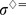
17.6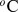. Moreover, removing the 6 worst predicted proteins reduces 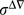 from 13.6 to 8.3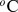. For comparison, we added in the Table the results obtained in direct validation, which yield a 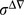 of 5.5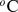.

**Table 1:** Values of the standard deviations 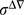 and 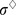 between the measured and the predicted melting temperatures (in degrees); 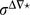 means the standard deviation excluding the 6 proteins whose 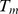 is predicted worst; 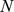 indicates the number of proteins in the family.

Family	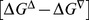	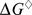	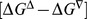	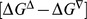	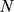
	jack knife	jack knife	jack knife	no jack knife	
	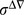	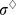	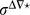	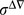	
Acylphosphatase	**7.5**	**25.2**	**4.7**	**3.0**	3
Ribonuclease	**17.3**	**23.0**	**3.5**	**2.7**	5
Lysozyme	**15.0**	**13.2**	**8.1**	**4.2**	4
Cell 12A endoglucanase	**13.7**	**9.6**	**5.4**	**4.4**	5
Adenylate kinase	**12.1**	**15.2**	**3.3**	**9.4**	6
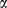 -Amylase	**7.5**	**9.5**	**7.6**	**4.2**	4
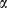 -Lactalbumin	**17.6**	**21.0**	**15.9**	**6.9**	3
Myoglobin	**19.9**	**19.4**	**15.4**	**7.8**	3
Cytochrome P450	**18.6**	**21.8**	**10.7**	**12.0**	5
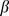 -Lactamase	**5.9**	**20.1**	**7.1**	**2.7**	4
Cold shock	**14.4**	**14.9**	**10.2**	**3.8**	3
Average	**13.6**	**17.6**	**8.3**	**5.5**	

The best predicted families are acylphosphatase, 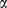-amylase and 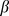-lactamase, with 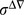-values between 5.9 and 7.5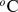, while the worst are cytochrome P450 and myoglobin, with 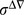-values around 19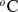. The proteins from the latter two families contain a heme, whereas the proteins from the other families contain no ligands or very small ones (see Tables S1–S11 in File S1). As our statistical potentials do not take into account the interactions with the ligands, mutations in the region of the heme are necessarily not estimated properly. The presence of the heme could thus well be the reason for the poor predictions in the cytochrome P450 and myoglobin families.

The average 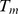 prediction score obtained with the standard, 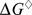-based, method is significantly lower than the one that uses 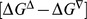. It is however noteworthy that some families are better predicted with the former method. This is clearly the case for the endoglucanase family and to a lower extent for the lysozyme family. This result suggests that these proteins are thermally stabilized through a shift of the entire stability curve towards lower 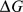-values.

## Discussion

A complete understanding of the features that determine protein thermal stability is still far from being reached. We have however made some progress towards this goal. The originality of our approach lies in the use of temperature-dependent statistical potentials, derived from distinct sets of protein structures, containing either mesostable or thermostable proteins. Linear combinations of these meso- and thermostable potentials, with coefficients identified so as to minimize the standard deviation between experimental and predicted 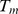
's, were used to predict the melting temperature on a set of 45 proteins that belong to 11 different homologous families.

These potentials allowed us to determine in an objective way the interactions that contribute most to protein stability in different temperature ranges and also, interestingly, the interactions that are less destabilizing - in other words, less repulsive - according to the temperature. For example, the temperature-dependent distance potentials point salt bridges, cation-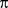 and aromatic interactions to contribute more to stability at high temperatures than hydrophobic packing, and conversely, and the interactions between positively charged residues to be less repulsive at high than at low temperature relative to other interactions [20], [21].

The novel temperature-dependent torsion potentials introduced here show also a significant dependence on the temperature. They provide indeed a non-negligible improvement of the 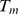 prediction performance. However, they are much more difficult to interpret in terms of specific interactions than distance potentials. Indeed, they reflect the propensities of amino acids and amino acid pairs to be associated to backbone torsion angle domains in their vicinity along the polypeptide chain, up to eight sequence positions further. These propensities are obviously related to secondary structure preferences but in an intricate way.

Another important feature that ensures the success of our approach is the focus on families of homologous proteins. We indeed defined family- and temperature-dependent statistical potentials, that include more proteins of the family under consideration and hence bias the potentials towards it. Note that we nevertheless kept the pairwise sequence similarity in the set to be at most 25%, to avoid uncontrolled biases. As the number of proteins with known 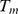 is quite limited, we also used proteins of unknown 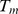 but of known 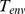 to enlarge the datasets from which potentials are derived, using three different rules to roughly estimate the former from the latter.

Note that the same approach as the one proposed here can be used for general 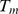 predictions, independently of protein families. However, this – as expected — decreases significantly the score of the predictions. On the other hand, we would like to emphasize that our method predicts the 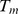 of a given protein from the 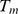 of homologous proteins, which have sometimes very different sequences. A much easier goal would be to predict the change in melting temperature upon point mutations (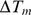).

The results presented here are very encouraging, but severely suffer from lack of data. Indeed, the number of proteins with experimentally determined structure and melting temperature is too limited, both for deriving sufficiently reliable temperature-dependent statistical potentials, and for biasing them properly towards a given protein family. The comparison of the score obtained in cross validation (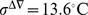 between predicted and measured 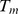
's) with the score in direct validation (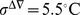) indicates that improvement can be expected from an increased dataset. Another source of errors is due to the fact that some families contain ligands, such as the hemes for the myoglobin and cytochrome families. These ligands sometimes strongly affect the stabilization properties of the proteins but cannot be taken into account in our potentials, which are limited to the residues of the polypeptide chain. This inevitably brings up the value of 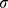. Finally, some experimental error should be included in the evaluation. This involves the intrinsic experimental error but, more importantly, the fact that the available experimental data are sometimes not performed exactly in the same experimental conditions in terms of pH, ionic strength, etc.

This discussion allows us to conclude on a positive note: the performance of our method is already quite good but is expected to significantly improve when larger datasets of proteins with known 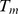, obtained in identical experimental conditions, will be available.

## Supporting Information

File S1Table S0, List of proteins with known melting temperature used in this study. Table S1–S11, List of proteins with known 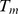 or 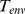 belonging to the 11 homologous families. Table S12, Experimental and predicted 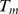
's of the proteins that belong to the 11 families. Table S13, Average melting temperature 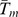 in the different datasets 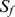. Table S14, Family-dependent 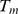-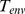 regression lines.(PDF)

## References

[1] Haki GD, Rakshit SK (2003) Developments in industrially important thermostable en-zymes: a review. Bioresour Technol 89: 17–34. 12676497 10.1016/s0960-8524(03)00033-6

[2] Bruins ME, Janssen AEM, Boom RM (2001) Thermozymes and their applications. Appl Biochem Biotechnol 90: 155–186. 11297390 10.1385/abab:90:2:155

[3] Frokjaer S, Otzen DE (2005) Protein drug stability: a formulation challenge. Nat Rev Drug Discov 4: 298–306. 15803194 10.1038/nrd1695

[4] de Carvalho CC (2011) Enzymatic and whole cell catalysis: finding new strategies for old processes. Biotechnol Adv 29: 75–83. 20837129 10.1016/j.biotechadv.2010.09.001

[5] Alcade M, Ferrer M, Plou FJ, Ballesteros A (2006) Environmental biocatalysis: from remediation with enzymes to novel green processes. Trends in Biotechnology 24: 281–287. 16647150 10.1016/j.tibtech.2006.04.002

[6] Mora M, Telford JL (2010) Genome-based approaches to vaccine development. Journal of Molecular Medicine 88: 143–147. 20066390 10.1007/s00109-009-0574-9

[7] Jaenicke R, Böhm G (1998) The stability of proteins in extreme environments. Current Opinion in Structural Biology 8: 738–748. 9914256 10.1016/s0959-440x(98)80094-8

[8] Vogt G, Woell S, Argos P (1997) Protein thermal stability, hydrogen bonds, and ion pairs. J Mol Biol 269: 631–43. 9217266 10.1006/jmbi.1997.1042

[9] Kumar S, Tsai CJ, Nussinov R (2001) Thermodynamic differences among homologous thermophilic and mesophilic proteins. Biochemistry 40: 14152–65. 11714268 10.1021/bi0106383

[10] Kumar S, Tsai CJ, Nussinov R (2000) Factors enhancing protein thermostability. Protein Eng 13: 179–91. 10775659 10.1093/protein/13.3.179

[11] Kumar S, Nussinov R (1999) Salt bridge stability in monomeric proteins. J Mol Biol 293: 1241–55. 10547298 10.1006/jmbi.1999.3218

[12] Kumar S, Nussinov R (2002) Close-range electrostatic interactions in proteins. Chem-biochem 3: 604–17. 10.1002/1439-7633(20020703)3:7<604::AID-CBIC604>3.0.CO;2-X 12324994

[13] Suhre K, Claverie JM (2003) Genomic correlates of hyperthermostability, an update. J Biol Chem 278: 17198–202. 12600994 10.1074/jbc.M301327200

[14] Thompson MJ, Eisenberg D (1999) Transproteomic evidence of a loop-deletion mecha-nism for enhancing protein thermostability. Journal of Molecular Biology 290: 595604. 10.1006/jmbi.1999.2889 10390356

[15] Chakravarty S, Varadarajan R (2002) Elucidation of factors responsible for enhanced thermal stability of proteins: a structural genomics based study. Biochemistry 41: 8152–61. 12069608 10.1021/bi025523t

[16] Berezovsky IN (2001) The diversity of physical forces and mechanisms in intermolecular interactions. Phys Biol 8: 035002. 10.1088/1478-3975/8/3/035002 21572170

[17] Ma BG, Goncearenco A, Berezovsky IN (2010) Thermophilic Adaptation of Protein Com-plexes Inferred from Proteomic Homology Modeling. Structure 18: 819–828. 20637418 10.1016/j.str.2010.04.004

[18] Elcock AH (1998) The stability of salt bridges at high temperatures: implications for hyperthermophilic proteins. J Mol Biol 284: 489–502. 9813132 10.1006/jmbi.1998.2159

[19] Berezovsky IN, Zeldovich KB, Shakhnovich EI (2007) Positive and Negative Design in Stability and Thermal Adaptation of Natural Proteins. PLoS Computational Biology 3: e52. 17381236 10.1371/journal.pcbi.0030052 PMC1829478

[20] Folch B, Dehouck Y, Rooman M (2010) Thermo- and mesostabilizing protein interactions identified by temperature-dependent statistical potentials. Biophys J 98: 667–77. 20159163 10.1016/j.bpj.2009.10.050 PMC2820637

[21] Folch B, Rooman M, Dehouck Y (2008) Thermostability of salt bridges versus hydropho-bic interactions in proteins probed by statistical potentials. J Chem Inf Model 48: 119–127. 18161956 10.1021/ci700237g

[22] Eijsink VG, Gaseidnes S, Borchert TV, Van den Burg B (2005) Directed evolution of enzyme stability. Biomol Eng 22: 21–30. 15857780 10.1016/j.bioeng.2004.12.003

[23] Counago R, Chen S, Shamoo Y (2006) In vivo molecular evolution reveals biophysical origins of organismal fitness. Mol Cell 22: 441–449. 16713575 10.1016/j.molcel.2006.04.012

[24] Wijma HJ, Floor RJ, Janssen DB (2013) Structure- and sequence-analysis inspired engi-neering of proteins for enhanced thermostability. Current Opinion in Structural Biology 23: 17. 10.1016/j.sbi.2013.04.008 23683520

[25] Korkegian A, Black ME, Baker D, Stoddard BL (2004) Computational Thermostabiliza-tion of an Enzyme. Science 308: 857–860. 10.1126/science.1107387 PMC3412875 15879217

[26] Shah PS, et al (2007) Full-sequence computational design and solution structure of a thermostable protein variant. J Mol Biol 372: 1–6. 17628593 10.1016/j.jmb.2007.06.032

[27] Seeliger D, de Groot BL (2010) Protein thermostability calculations using alchemical free energy simulations. Biophys J 98: 2309–16. 20483340 10.1016/j.bpj.2010.01.051 PMC2872215

[28] Bae E, Bannen RM, Phillips GN Jr (2008) Bioinformatic method for protein thermal stabilization by structural entropy optimization. Proc Natl Acad Sci U S A 105: 9594–7. 18621726 10.1073/pnas.0800938105 PMC2474475

[29] Chan CH, Liang HK, Hsiao NW, Ko MT, Lyu PC, et al (2004) Relationship between local structural entropy and protein ther-mostabilty. Proteins: Structure, Function, and Bioinformatics 57: 684–691. 10.1002/prot.20263 15532068

[30] Ku T, Lu P, Chan C, Wang T, Lai S, et al (2009) Predicting melting temperature directly from protein sequences. Computational Biology and Chemistry 33: 445–450. 19896904 10.1016/j.compbiolchem.2009.10.002

[31] Potapov V, Cohen M, Schreiber G (2009) Assessing computational methods for predicting protein stability upon mutation: good on average but not in the details. Protein Eng Des Sel 2: 553–556. 10.1093/protein/gzp030 19561092

[32] Dehouck Y, Grosfils A, Folch B, Gilis D, Bogaerts Ph, et al (2009) Fast and ac-curate predictions of protein stability changes upon mutations using statistical potentials and neural networks: PoPMuSiC-2.0. Bioinformatics 25: 2537–2543. 19654118 10.1093/bioinformatics/btp445

[33] Khan S, Vihinen M (2010) Performance of protein stability predictors. Hum Mutat 3: 675–684. 10.1002/humu.21242 20232415

[34] Li Y, Fang J (2012) PROTS-RF: a robust model for predicting mutation-induced protein stability changes. PLoS One 7: e47247. 23077576 10.1371/journal.pone.0047247 PMC3471942

[35] Dehouck Y, Gilis D, Rooman M (2006) A new generation of statistical potentials for proteins. Biophys J 90: 40104017. 10.1529/biophysj.105.079434 PMC1459517 16533849

[36] Kumar MD, Bava KA, Gromiha MM, Prabakaran P, Kitajima K, et al (2006) ProTherm and ProNIT: thermodynamic databases for proteins and protein-nucleic acid interactions. Nuleic Acids Res 34: D204–6. 10.1093/nar/gkj103 PMC1347465 16381846

[37] Wang G, Dunbrack RL Jr (2003) PISCES: a protein sequence culling server. Bioinfor-matics 19: 1589–1591. 10.1093/bioinformatics/btg224 12912846

[38] Gromiha MM, Oobatake M, Sarai A (1999) Important amino acid properties for enhanced thermostability from mesophilic to thermophilic proteins. Biophys Chem 82: 51–67. 10584295 10.1016/s0301-4622(99)00103-9

[39] Dehouck Y, Folch B, Rooman M (2008) Revisiting the correlation between proteins' thermoresistance and organisms' thermophilicity. Protein Eng Des Sel 21: 275–8. 18245807 10.1093/protein/gzn001

[40] Pearson WR, Lipman DJ (1988) Improved tools for biological sequence comparison. Proc Natl Acad Sci U S A 85: 2444–8. 3162770 10.1073/pnas.85.8.2444 PMC280013

[41] Rooman M, Kocher JP, Wodak SJ (1991) Prediction of backbone conformation based on seven structure assignments. Influence of local interactions, J Mol Biol 221: 961–979. 1942039 10.1016/0022-2836(91)80186-x

[42] Gromiha MM (2001) Important inter-residue contacts for enhancing the thermal stability of thermophilic proteins. Biophys Chem 91: 71–7. 11403885 10.1016/s0301-4622(01)00154-5

[43] Kannan N, Vishveshwara S (2000) Aromatic clusters: a determinant of thermal stability of thermophilic proteins. Protein Eng 13: 753–61. 11161106 10.1093/protein/13.11.753

[44] Nojima H, Hon-Nami K, Oshima T, Noda H (1978) Reversible thermal unfolding of thermostable cytochrome c-552. J Mol Biol 122: 33–42. 209196 10.1016/0022-2836(78)90106-7

[45] Razvi A, Scholtz JM (2006) Lessons in stability from thermophilic proteins. Protein Sci 15: 1569–1578. 16815912 10.1110/ps.062130306 PMC2242557

