# Supplementary material for: Protein Thermostability Prediction within Homologous Families Using Temperature-Dependent Statistical Potentials
Source: PLoS One. 2014 Mar 19;9(3):e91659. doi: 10.1371/journal.pone.0091659 (PMC3960129; doi:10.1371/journal.pone.0091659)
Supplement: File S1 — Table S0, List of proteins with known melting temperature used in this study. Table S1–S11, List of proteins with known or belonging to the 11 homologous families. Table S12, Experimental and predicted 's of the proteins that belong to the 11 families. Table S13, Average melting temperature in the different datasets . Table S14, Family-dependent - regression lines. (PDF) [file pone.0091659.s001.pdf]

Supporting Information

Protein thermostability prediction within homologous families using temperature-dependent statistical potentials

Fabrizio Pucci, Malik Dhanani, Yves Dehouck and Marianne Rooman

- Table S0 : List of proteins with known melting temperature used in this study.
- Table S1-S11 : List of proteins with known T_m or T_{env} belonging to the 11 homologous families.
- Table S12 : Experimental and predicted T_m's of the proteins that belong to the 11 families.
- Table S13 : Average melting temperature $\bar{T}_m$ in the different datasets S_f.
- Table S14 : Family-dependent T_m-T_{env} regression lines.
- References

PDB code	*Tm exp (°C)*	pH	Protein name	Resolution (Å)	Host organism	Number of residues	Reference(s)
1fga	39.45	6.60	Fibroblast growth factor	2.20	*Human (homo sapiens)*	124	BIOCHEMISTRY 39, 7153-7158 (2000);
1hml	39.50	7.00	Alpha lactalbumin	1.70	*Human (homo sapiens)*	123	BIOCHEMISTRY 28, 8568-8576 (1989);
1rg8	40.25 (0.45)	7.30	Acidic fibroblast growth factor	1.10	*Human (homo sapiens)*	141	PROTEIN ENG DES SEL 17, 603-611 (2004);
1ihb	41.53	7.50	Cyclin-dependent kinase inhibitor	1.95	*Human (homo sapiens)*	156	J BIOL CHEM 277, 48827-48833 (2002);
1blc	41.60	7.50	Beta lactamase	2.20	*Staphylococcus aureus*	257	BIOCHEMISTRY 33, 116-125 (1994);
1d0b	42.10	7.50	Internalin b	1.86	*Listeria monocytogenes*	207	J MOL BIOL 337, 453-461 (2004);
1chk	43.20	7.00	Chitosanase	2.40	*Streptomyces lividans*	238	BIOCHIM BIOPHYS ACTA 1429, 365-376 (1999);
1p3j	43.30	7.40	Adenylate kinase	1.90	*Bacillus subtilis*	212	J BIOL CHEM 279, 28202-28208 (2004);
1aqh	44.00	7.20	Alpha-amylase	2.00	*Alteromonas haloplanctis*	448	J BIOL CHEM 276 25791-25796 (2001);
1rtp	45.80	7.40	Parvalbumin	2.00	*Bos taurus*	109	FEBS LETT 442, 241-245 (1999);
1ttq	46.00	7.80	Tryptophan synthase alpha subunit	2.00	*Salmonella typhimurium*	256	BIOCHEM BIOPHYS RES COMMUN 151, 672-678 (1988);
1orc	46.40	7.00	Cro repressor	1.54	*Bacteriophage lambda*	64	FEBS LETT 289 201-204 (1991);
1efc	46.50	7.60	Ef-tu elongation factor	2.02	*Escherichia coli*	386	PROTEIN SCI 13, 89-99 (2004);
2ci2	46.60	6.30	Chymotrypsin inhibitor	2.00	*Hordeum vulgare*	65	BIOCHEMISTRY 34, 1695-1701 (1995);
1bu7	47.00	7.40	Cytochrome p450	1.65	*Bacillus megaterium*	455	J BIOL CHEM 278, 608-616 (2003);
1s3g	47.60	7.40	Adenylate kinase	2.25	*Bacillus globisporus*	217	J BIOL CHEM 279, 28202-28208 (2004);
1aky	47.70	7.50	Adenylate kinase	1.96	*Saccharomyces cerevisiae*	218	EUR J BIOCHEM 231, 405-413 (1995);
1mve	48.00	7.00	Truncated glucanase	1.70	*Fibrobacter succinogenes*	238	BIOCHEMISTRY 44, 9197-9205 (2005);
1lni	48.16 (0.61)	7.00	Ribonuclease sa	1.00	*Streptomyces aureofaciens*	96	BIOCHEMISTRY 37, 16192-16200 (1998);PROTEIN SCI 8, 1843-1849 (1999);J BIOL CHEM 278, 31790-31795 (2003);PROTEIN SCI 12, 2367-2373 (2003);J MOL BIOL 354, 967-978 (2005);
1oa3	49.20	8.00	Cel12a	1.70	*Hypocrea schweinitzii*	217	PROTEIN SCI 12, 848-860 (2003);
1rn1	49.55 (1.25)	7.00	Ribonuclease t1	1.84	*Aspergillus oryzae*	104	J BIOL CHEM 264, 11621-11625 (1989);;
2cpp	50.15	7.40	Cytochrome p450	1.63	*Pseudomonas putida*	405	BIOCHEMISTRY 45, 12715-12722 (2006);
1rx4	50.20	7.80	Dihydrofolate reductase	2.20	*Escherichia coli*	159	J BIOL CHEM 282, 9420-9429 (2007);
1kfw	50.50	7.50	Chitinase	1.74	*Arthrobacter sp.*	435	PROTEIN ENG 16, 497-503 (2003);
1cpm	50.80	6.00	Glucanase	2.00	*Paenibacillus macerans*	214	PROTEIN SCI 5, 2255-2265 (1996);
1pii	51.00	7.40	Anthranilate isomerase	2.00	*Escherichia coli*	452	BIOCHEMISTRY 33, 6350-6355 (1994);
1bmc	51.03	7.00	Beta lactamase	2.50	*Bacillus cereus*	213	BIOCHEMISTRY 31, 6603-6607 (1992);
1wlg	51.10	7.00	Flagellar hook protein	1.80	*Salmonella typhimurium*	293	J MOL BIOL 251, 520-532 (1995);
1ayf	51.39	8.50	Adrenodoxin	1.85	*Bos taurus*	102	PROTEIN SCI 5, 1890-1897 (1996);
1ank	51.80	7.20	Adenylate kinase	2.00	*Escherichia coli*	214	J BIOL CHEM 266, 23654-23659 (1991);
1bd8	51.90	7.50	Cyclin-dependent kinase inhibitor p19	1.80	*Human (homo sapiens)*	156	J MOL BIOL 315 447-457 (2002);
2fal	52.00	7.00	Myoglobin	1.80	*Sea hare (aplysia limacina)*	146	J MOL BIOL 297, 1231-1244 (2000);
1ew4	52.07 (1.29)	7.00	Frataxin cyay	1.40	*Escherichia coli*	106	J MOL BIOL 336, 203-212 (2004);BIOCHEMISTRY 43, 6511-6518 (2004);
9rnt	52.10	7.00	Rnase t1	1.50	*Aspergillus oryzae*	104	BIOCHEMISTRY 33, 3312 (1994);
1am7	52.30	7.00	Lysozyme	2.30	*Bacteriophage lambda*	150	FEBS LETT 460, 442-446 (1999);
1avr	52.50	8.00	Annexin v	2.30	*Human (homo sapiens)*	317	BIOCHEMISTRY 36, 1657-1668 (1997);
1h12	52.60	7.50	Xylanase 8	1.20	*Pseudoalteromonas haloplanktis*	404	J BIOL CHEM 277, 35133-35139 (2002);
2fx5	53.00	5.80	Lipase	1.80	*Pseudomonas mendocina*	258	PROTEIN SCI 15, 1915-1927 (2006) PMID: 16823035;
2nvh	53.00	3.00	Interleukin 1 beta	1.53	*Human (homo sapiens)*	153	BIOCHEMISTRY 33, 9327-9332 (1994);
2rn2	53.10	5.50	Ribonuclease hi	1.48	*Escherichia coli*	155	EUR J BIOCHEM 220, 623-631 (1994);
5pep	53.10 (0.50)	8.00	Pepsin	2.34	*Porcine (sus scrofa)*	326	BIOCHEMISTRY 39, 4182-4190 (2000);
1ey0	53.15 (0.15)	7.00	Staphylococcal nuclease	1.60	*Staphylococcus aureus*	136	PROTEIN SCI 4, 2545-2558 (1995);J MOL BIOL 303, 125-130 (2000);
1abe	53.57	7.40	Arabinose binding protein	1.70	*Escherichia coli*	305	J BIOL CHEM 258, 13193-13198 (1983);
1rro	53.60	7.40	Beta-parvalbumin	1.30	*Rat (rattus rattus)*	108	FEBS LETT 442, 241-245 (1999);
1yea	53.63 (0.90)	6.00	Iso-2 cytochrome c	1.90	*Saccharomyces cerevisiae*	112	BIOCHEMISTRY 33, 9209-9219 (1994);BIOCHEMISTRY 35, 1995-2007 (1996); PROTEIN SCI 9, 536-543 (2000);
1e21	53.70	5.00	Ribonuclease a	1.90	*Human (homo sapiens)*	119	PROTEIN ENG 15, 887-893 (2002);
2acy	53.80	5.50	Acylphosphatase	1.80	*Bos taurus*	98	PROTEINS 62, 64-79 (2006);

1csp	53.80	7.00	Cold shock protein	2.50	*Bacillus subtilis*	67	J MOL BIOL 347, 1063-1076 (2005);
1hxn	53.90	7.40	Hemopexin	1.80	*Oryctolagus cuniculus*	209	BIOCHEMISTRY 32, 7216-7222 (1993);
1bni	54.00	7.00	Barnase	2.10	*Bacillus amyloliquefaciens*	108	BIOCHEMISTRY 34, 5224-5233 (1995);
1h8v	54.50	8.00	Cel12a	1.90	*Trichoderma reesei*	217	PROTEIN SCI 12, 2782-2793 (2003);
1ke4	54.60	6.80	Beta lactamase	1.72	*Escherichia coli*	357	PROTEIN SCI 8, 1816-1824 (1999);
1oxa	55.00	7.40	Cytochrome p450	2.10	*Saccarapolyspora erythraea*	403	J BIOL CHEM 278, 608-616 (2003);
3pgk	56.15	7.00	Phosphoglycerate kinase	2.50	*Saccharomyces cerevisiae*	415	BIOCHEMISTRY 28, 813-818 (1989);
1hfz	56.20	7.40	Alpha lactalbumin	2.30	*Bos taurus*	123	PROTEIN ENG 12, 581-587 (1999);
2rbi	56.50	6.20	Binase	2.20	*Bacillus intermedius*	108	FEBS LETT 445, 384-388 (1999);
1mjc	56.94 (0.99)	7.00	Cold shock protein	2.00	*Escherichia coli*	69	PROTEIN SCI 9, 387-394 (2000);PROTEIN SCI 10 2028-2036 (2001);
3sil	57.00	7.00	Sialidase	1.05	*Salmonella typhimurium*	381	PROTEIN ENG 14 891-896 (2001);
1ten	57.10	5.00	Tenascin	1.80	*Human (homo sapiens)*	90	BIOCHEMISTRY 37, 8071-8079 (1998);
1lmb	57.20	7.00	Lambda repressor	1.70	*Phage lambda*	80	BIOCHEMISTRY 41, 5359-5374 (2002);
1ftg	57.35 (0.05)	7.00	Apoflavodoxin	2.00	*Anabaena PCC7119*	168	PROTEIN SCI 5, 1376-1388 (1996);
3psg	57.50	7.10	Pepsinogen	1.65	*Porcine (sus scrofa)*	365	J MOL BIOL 152 445-464 (1981);
1tca	57.70	7.00	Lipase b	1.55	*Candida antarctica*	317	PROTEIN ENG 16, 599-605 (2003);
1pmk	57.80	7.00	Plasminogen kringle 4 domain	2.25	*Human (homo sapiens)*	78	BIOCHEMISTRY 32, 8799-8806 (1993);
1v0s	57.80	5.50	Phospholipase d	1.75	*Streptomyces septatus*	495	BIOCHIM BIOPHYS ACTA 1696, 75-82 (2004);
1qlp	58.87 (0.66)	7.80	Alpha1-antitrypsin	2.00	*Human (homo sapiens)*	372	J MOL BIOL 313 1161-1169 (2001);BIOCHEMISTRY 41, 4575-4581 (2002);J MOL BIOL 325, 581-589 (2003);
1avu	59.00	7.00	Kunitz type soybean trypsin inhibitor (sti)	2.30	*Glycine max*	172	BIOCHEMISTRY 41, 5359-5374 (2002);
2cab	59.00	6.10	Carbonic hydrase b	2.00	*Human (homo sapiens)*	256	BIOCHEMISTRY 44, 5258-5266 (2005);
2a01	59.20 (0.75)	7.40	Apolipoprotein a-i	2.40	*Human (homo sapiens)*	243	J MOL BIOL 378, 264-272 (2008);BIOCHEMISTRY 39, 15910-15919 (2000); BIOCHEMISTRY 41, 10529-10539 (2002);BIOCHEMISTRY 45, 1242-1254 (2006);
1wq5	59.50	7.20	Tryptophan synthase alpha subunit	2.30	*Escherichia coli*	258	ARCH BIOCHEM BIOPHYS 292, 34-41 (1992);
1ekg	60.00	7.00	Frataxin	1.80	*Human (homo sapiens)*	119	BIOCHEMISTRY 43, 6511-6518 (2004);
1ycc	60.00	6.00	Iso-1 cytochrome c	1.23	*Saccharomyces cerevisiae*	108	PROTEIN SCI 8, 2645-2654 (1999);
1rhg	60.95	6.90	Granulocyte colony stimulating factor	2.20	*Human (homo sapiens)*	145	BIOCHEMISTRY 41, 6422-6431 (2002);
1akd	61.00	7.40	Cytochrome p450	1.80	*Pseudomonas putida*	405	J BIOL CHEM 278, 608-616 (2003);
1w2p	61.20	7.00	Xylanase 10	1.45	*Cellvibrio japonicus*	346	J MOL BIOL 279, 54369-54379 (2004);
1xyp	61.40	5.00	Xylanase ii	1.50	*Trichoderma reesei*	189	J BIOTECHNOL 108, 137-143 (2004);
1esf	61.40	7.00	Enterotoxin a	1.90	*Staphylococcus aureus*	229	J BIOL CHEM 275, 1665-1672 (2000);
6taa	62.00	7.00	Taka-amylase	2.10	*Aspergillus oryzae*	476	BIOCHEMISTRY 26, 4063-4068 (1987);
1shg	62.00	7.00	Sh3 spectrin	1.80	*Escherichia coli*	57	J MOL BIOL 357, 1592-1604 (2006) PMID: 16487539;
1qwo	62.50	5.00	Phytase atcc	1.50	*Aspergillus fumigatus*	435	PROT ENG 13, 49-57 (2000);
1rtb	62.80	7.10	Ribonuclease a	2.50	*Bos taurus*	124	BIOCHEMISTRY 45, 10795-10806 (2006);
1poh	63.40	7.00	Histidine-containing phosphocarrier protein	2.00	*Escherichia coli*	85	J MOL BIOL 286, 1609-1619 (1999);
3rn3	63.60	7.00	Rnase a	1.45	*Bos taurus*	124	PROTEIN SCI 8, 832-840 (1999);
1tpk	64.30	4.50	Tissue plasminogen activator	2.40	*Human (homo sapiens)*	88	BIOCHEMISTRY 28, 4047-4054 (1989);
1bk7	64.40	7.50	Rnase mc1	1.75	*Momordica charantia (seeds bitter)*	190	BIOSCI BIOTECHNOL BIOCHEM 68, 1748-1757 (2004);
1fna	64.40	7.40	Fibronectin (10th type iii module)	1.8	*Human (homo sapiens)*	91	J MOL BIOL 217, 563-575 (1991);
3mbp	64.60	7.00	Maltose-binding protein	1.70	*EScherichia coli*	370	PROTEINS 53, 863-871 (2003);
1wdn	64.65 (1.55)	8.30	Glutamine-binding protein	1.94	*Escherichia coli*	223	PROTEINS 58, 80-87 (2005);PROTEINS 58, 80-87 (2005);
2lzm	64.85 (0.15)	6.50	Lysozyme	1.70	*Escherichia coli*	164	NATURE 334, 406-410 (1988);PROC NATL ACAD SCI U S A 85, 401-405 (1988);
1lz1	64.90	2.70	Lysozyme	1.50	*Human (homo sapiens)*	130	J MOL BIOL 254, 62-76 (1995);
1sup	65.00	7.00	Subtilisin bpn	1.60	*Bacillus amyloliquefaciens*	275	J BIOL CHEM 277, 27553-27558 (2002);
4blm	65.00	7.00	Beta lactamase	2.00	*Bacillus licheniformis*	256	BIOCHEMISTRY 29, 5797-5806 (1990);
1tgn	65.50 (0.50)	4.20	Trypsinogen	1.65	*Bos taurus*	222	J MOL BIOL 247, 701-716 (1995);
1ppi	65.60	7.20	Alpha-amylase	2.20	*Porcine (sus scrofa)*	496	J BIOL CHEM 276 25791-25796 (2001);
1jae	65.90	7.20	Alpha-amylase	1.65	*Tenebrio molitor*	470	BIOCHEMISTRY 38, 4613-4619 (1999);
1g6n	66.40	7.00	Catabolite activator protein	2.1	*Escherichia coli*	200	BIOCHEMISTRY 27, 5257-5261 (1988);
1oa4	66.80	8.00	Cel12a	1.50	*Streptomyces sp. 11ag8*	222	PROTEIN SCI 12, 848-860 (2003);
1gwy	67.00	7.50	Sticholysin	1.71	*Stoichactis helianthus*	175	FEBS LETT 575, 14-18 (2004);

1cyo	67.10 (0.44)	7.00	Cytochrome b5	1.50	*Bos taurus*	88	PROTEIN ENG 10, 575-581 (1997);BIOCHEMISTRY 38, 11961-11972 (1999);BIOPHYS CHEM 83, 3-17 (2000);PROTEIN ENG 6, 953-964 (1993);FEBS LETT 314, 419-424 (19992);
1cea	67.60	7.40	Plasminogen (kringle 1 domain)	2.10	*Human (homo sapiens)*	80	BIOCHEMISTRY 30, 1948-1957 (1991);
2hip	67.70	7.00	High-potential iron sulfur protein (hipip)iso-i	2.50	*Ectothiorhodospira halophila*	71	PROTEIN SCI 4, 2562-2572 (1995);
4tln	68.00	7.50	Thermolysin	2.30	*Bacillus thermoproteolyticus*	316	BIOCHEMISTRY 35, 3477-3486 (1996);
1osa	68.54	7.40	Calmodulin	1.68	*Paramecium*	148	PROTEINS 50, 381-391 (2003);
1olr	68.70	8.00	Cel12a	1.20	*Humicola grisea*	223	PROTEIN SCI 12, 2782-2793 (2003);
1znj	68.70	7.40	Insulin	2.00	*Human (homo sapiens)*	30	BIOCHEMISTRY 44, 11171-11177 (2005);BIOCHEMISTRY 45, 4014-4024 (2006);
1rbp	68.90	7.40	Serum retinol binding protein	2.00	*Human (homo sapiens)*	175	PROTEIN SCI 10 2301-2316 (2001);
1col	69.00	7.00	Colicin a	2.4	*Escherichia coli*	197	J BIOL CHEM 268, 1553-1557 (1993);
1ji3	70.00	7.00	Lipase l1	2.20	*Bacillus stearothermophilus*	388	BIOSCI BIOTECHNOL BIOCHEM 64, 280-286 (2000);
1uw3	70.00	7.50	Prion protein	2.04	*Ovis aries*	106	BIOCHEMISTRY 41, 11017-11024 (2002);
1a70	70.00	7.00	Ferredoxin	1.7	*Spinacia oleracea*	97	BIOCHEMISTRY 42, 1354-1364 (2003);
2imm	70.20	7.00	Immunoglobulin	2.00	*Mus musculus*	114	BIOCHEMISTRY 30, 6922-6929 (1991);
1smd	70.30	7.20	Alpha-amylase	1.60	*Human (homo sapiens)*	495	BIOCHEMISTRY 38, 4613-4619 (1999);
1cec	70.40	7.20	Cellulase c	2.15	*Clostridium thermocellum*	331	BIOPHYS CHEM 132, 229-241 (2002);
1hmk	70.78 (0.47)	7.50	Alpha-lactalbumin	2.00	*Capra hircus*	121	PROTEINS 60, 118-130 (2005);PROTEINS 60, 118-130 (2005);PROTEINS 60, 118-130 (2005);PROTEINS 60, 118-130 (2005);PROTEINS 60, 118-130 (2005);
1pca	71.00	7.50	Carboxypeptidase a	2.00	*Porcine (sus scrofa)*	403	EUR J BIOCHEM 176, 225-230 (1988);
1ag6	71.00	7.00	Plastocyanin	1.60	*Spinacia oleracea*	99	BIOCHEMISTRY 42, 10301-10310 (2003);
4gcr	72.00	6.80	Gamma-crystallin	1.47	*Bos taurus*	174	J BIOL CHEM 268, 18119-18127 (1993);
1tml	72.37 (0.17)	6.80	Cellulase e2	1.80	*Thermomonospora fusca*	286	BIOCHEMISTRY 38, 2570-2576 (1999);
2hpr	73.00	7.00	Hpr protein	2.00	*Bacillus subtilis*	87	BIOCHEMISTRY 45, 4084-4092 (2006);
1jbk	73.00	7.50	Cplb	1.80	*Escherichia coli*	189	PROTEIN SCI 11, 1192-1198 (2002);
1zip	74.50	7.20	Adenylate kinase	1.85	*Bacillus stearothermophilus*	217	BIOCHEMISTRY 31, 3038-3043 (1992);
4lyz	74.80	7.00	Lysozyme	2.00	*Gallus gallus*	129	PROTEINS 40, 49-57 (2000);
1svn	75.50	9.00	Savinase	1.40	*Bacillus lentus*	269	PROT ENG 17, 149-156 (2004);
1fvk	76.77	7.00	Dsba	1.7	*Escherichia coli*	188	PROTEIN SCI 8, 106-112 (1999);
1c9o	76.90	7.00	Cold shock protein	1.17	*Bacillus caldolyticus*	66	NAT STRUCT BIOL 7, 380-383 (2000);J MOL BIOL 319, 541-554 (2002);PROTEIN ENG DES SEL 19, 355-358 (2006) PMID: 16720692;
1aye	77.00	7.00	Ada2h	1.80	*Human (homo sapiens)*	401	BIOCHEMISTRY 41, 5359-5374 (2002);
1poo	77.60	7.00	Phytase	2.10	*Bacillus amyloliquefaciens*	353	NATURE 7, 147-153 (2000);
1ymb	78.30	7.00	Myoglobin	1.90	*Equus caballus*	153	BIOCHEMISTRY 40 5075-5080 (2001);
1ova	78.40	7.00	Ovalbumin	1.95	*Gallus gallus*	385	PROTEIN SCI 12, 2693-2703 (2003);
1gtg	79.40	5.50	Kumamolisin	2.3	*Bacillus novosp. mn-32*	357	BIOCHIM BIOPHYS ACTA 1764, 364-371 (2006);
1pgx	79.40	5.40	B2 of protein g	1.50	*Streptococcus*	70	BIOCHEMISTRY 31 3597 (1992);
1shf	80.10	8.00	Fyn tyrosine kinase (sh3 domain)	1.9	*Human (homo sapiens)*	59	BIOCHEMISTRY 37, 16172-16182 (1998);J MOL BIOL 333, 641-655 (2003);
1e65	81.00	7.03	Azurin	1.85	*Pseudomonas aeruginosa*	128	J PHYS CHEM 99 14864-14870 (1995);
1hrc	81.60	6.00	Cytochrome c	1.90	*Equus caballus*	104	BIOCHIM BIOPHYS ACTA 1646, 49-56 (2003);
1hgu	82.00	5.50	Human growth hormone	2.50	*Human (homo sapiens)*	187	PROTEIN SCI 11, 1452-1461 (2002);
4mbn	82.20	9.60	Myoglobin	2.00	*Physeter catodon*	153	PROTEIN SCI 2, 1099-1105 (1993);
1bvc	82.20	9.60	Myoglobin	1.5	*Cetacea sperm*	153	PROTEIN SCI 2, 1099-1105 (1993);
9pap	83.00	5.60	Papain	1.65	*Papaya (carica papaya)*	212	INT J BIOL MACROMOL 41, 383-390 (2007);
2ocj	84.80	7.00	Tumor suppressor p53	2.05	*Human (homo sapiens)*	194	BIOCHEMISTRY 34, 5309-5316 (1995);
1io2	85.20	9.00	Ribonuclease hii	2.00	*Thermococcus kodakaraensis*	213	BIOCHEMISTRY 45, 12673-12679 (2006);
2ovo	85.20	4.51	Ovomucoid third domain	1.50	*Silver pheasant (lophura nycthemera)*	56	BIOCHEMISTRY 34, 4724-4732 (1995);
2trx	85.46 (1.85)	7.00	Thioredoxin	1.68	*Escherichia coli*	108	BIOCHEMISTRY 34, 2148-2152 (1995);BIOCHEMISTRY 26, 1406-1411 (1987); BIOCHEMISTRY 31, 4901-4907 (1992);BIOCHEMISTRY 32, 7526-7530 (1993); BIOCHEMISTRY 40 10047-10053 (2001);
1cse	85.83	7.00	Eglin c	1.2	*Hirudo medicinalis*	63	BIOPHYS CHEM 55, 247-252 (1995);
1iv7	85.85	7.00	Single chain monellin (scm)	1.82	*Dioscoreophyllum cumminisii*	96	J BIOL CHEM 276, 19624-19630 (2001);
1bsq	86.00	7.00	Beta-lactoglobulin type b	2.22	*Bos taurus*	162	INT J BIOL MACROMOL 38, 9-17 (2006);

1ppn	86.20	3.90	Proteinase	1.6	*Carica papaya*	212	EUR J BIOCHEM 214, 129-134 (1993);
1onc	87.80	7.00	Onconase	1.70	*Rana pipiens*	103	BIOCHEMISTRY 39, 8711-8718 (2000);
1n97	88.00	7.40	Cytochrome p450	1.80	*Thermus thermophilus*	385	J BIOL CHEM 278, 608-616 (2003);
1pga	88.00	7.20	Protein g	2.07	*Streptococcus*	56	BIOCHEMISTRY 39, 965-977 (2000);
1i4n	90.30	7.50	Indole glycerol phosphate synthase	2.50	*Thermotoga maritima*	251	J MOL BIOL 288, 753-763 (1999);
1azp	90.70	7.00	Sac7d	1.60	*Sulfolobus acidocaldarius*	66	BIOCHEMISTRY 43, 2840-2853 (2004);BIOCHEMISTRY 44, 915-925 (2005);
1f4t	91.00	7.40	Cytochrome p450	1.93	*Sulfolobus solfataricus*	367	J BIOL CHEM 278, 608-616 (2003);
1ubq	91.00	4.00	Ubiquitin	1.80	*Human (homo sapiens)*	76	PROTEINS 18, 246-253 (1994);
1h7m	93.65 (0.15)	7.40	Ribosomal protein l30e	1.96	*Thermococcus celer*	98	PROTEIN SCI 12, 1483-1495 (2003);J MOL BIOL 348, 419-431 (2005);
1jji	99.00	7.50	Esterase	2.20	*Archaeoglobus fulgidus*	311	BIOCHEMISTRY 41 1364-1371 (2002);
2bjd	100.80	5.50	Acylphosphatase	1.27	*Sulfolobus solfataricus*	90	PROTEINS 62, 64-79 (2006);
1tmy	100.85	6.00	Chey	1.90	*Thermotoga maritima*	118	BIOCHEMISTRY 40 13107-13113 (2001);
1bli	101.00	7.40	Alpha-amylase	1.90	*Bacillus licheniformis*	481	BIOCHEMISTRY 43, 9589-9599(2004);
1r0f	104.00	7.50	Rubredoxin	1.60	*Clostridium pasteurianum*	53	PROTEINS 57, 118-127 (2004);
5pti	104.00	7.00	Trypsin inhibitor	1.00	*Bos taurus*	58	BIOCHEMISTRY 36, 5323-5335 (1997);
2a3m	108.00	7.60	Cytochrome g20c3	1.50	*Desulfovibrio desulfuricans*	107	J MOL BIOL 358, 1-14 (2006);
2d0s	108.00	5.00	Cytochrome c 552	2.20	*Hydrogenophilus thermoluteolus*	79	BIOCHEMISTRY 45, 6115-6123 (2006);
451c	109.00	7.00	Cytochrome c551	1.6	*Pseudomonas aeruginosa*	82	J AM CHEM SOC 126, 14684-14685 (2004);
1w2i	111.50	7.40	Acylphosphatase	1.50	*Pyrococcus horikoshii*	90	BIOCHEMISTRY 44, 4601-4611 (2005);
2cth	121.60	7.60	Cytochrome c3	1.67	*Desulfovibrio vulgaris*	107	BIOCHEMISTRY 38, 33-41 (1999);
1brf	143.80	7.00	Rubredoxin	1.62	*Pyrococcus furiosus*	53	PROTEINS 57, 118-127 (2004);

Table S 0. List of proteins with known melting temperature used in this study.

The number in parenthesis in the experimental Tm column corresponds to the standard deviation when there are different experimental values.

α-Amylase

PDB code	Host Organism	T_m^{exp} (°C)	T_{env} (°C)	$T_m^{(1)\,est}$ (°C)	$T_m^{(2)\,est}$ (°C)	$T_m^{(3)\,est}$ (°C)	Resol. ($\AA$)	Number of residues	Ligand [1]	Ref.
**1aqh**	Pseudoalteromonas haloplanktis	44.0	26.0				2.00	453		[2]
1pig	Sus scrofa		39.0	67.1	71.0	44.0	2.20	496	x	[3]
**1smd**	Homo sapiens	70.0	37.0				1.60	496		[4]
**1ppi**	Homo sapiens	65.6	37.0				2.20	496	x	[2]
**1jae**	Tenebrio molitor	65.9	28.0				1.65	471		[4]
1ua7	Bacillus subtilis		37.0	65.9	67.7	44.0	2.21	422	x	[5]
3dc0	Bacillus sp. KR-8104		50.0	73.9	89.2	44.0	2.78	422		[6]
2guy	Aspergillus oryzae		34.0	64.0	62.8	65.6	1.59	478	x	[7]
1cxi	Bacillus circulans		30.0	61.5	56.2	70.0	2.00	686	x	[5]
1qh0	Geobacillus stearothermophilus		51.0	74.5	90.8	44.0	1.70	686	x	[8]
3bh4	Bacillus amyloliquefaciens		35.0	64.6	64.4	44.0	1.40	483		[9]
2die	Bacillus Sp. Ksm-1378		33.0	63.4	61.1	65.9	2.10	485		[10]
3bcf	Halothermothrix orenii H 168		60.0	80.1	105.7	70.0	2.30	599		[11]
1bvz	Thermoactinomyces vulgaris		55.0	77.0	97.4	65.9	2.60	585		[5]
2aaa	Aspergillus niger		30.0	61.5	56.2	44.0	2.10	484		[12]
1ava	Hordeum vulgare		23.0	57.2	44.6	44.0	1.90	403		[13]
2gjr	Bacillus halmapalus		30.0	61.5	56.17	70.0	2.10	485	x	[14]
1mwo	Pyrococcus woesei		101.5	105.8	174.1	65.6	2.20	435		[15]
3m07	Salmonella typhimurium		37.0	65.9	67.7	70.0	1.40	618	x	[16]

Table S 1: Proteins of the α-Amylase family.

Lysozyme

PDB code	Host Organism	T_m^{exp} (°C)	T_{env} (°C)	$T_m^{(1)\text{ est}}$ (°C)	$T_m^{(2)\text{ est}}$ (°C)	$T_m^{(3)\text{ est}}$ (°C)	Resol. (Å)	Number of residues	Ligand [1]	Ref.
**1am7**	Enterobacteria phage lambda	52.2	37.0				2.30	158	x	[17]
2z2f	Bos taurus		38.6	66.8	68.7	64.9	1.50	129	x	[18]
**2lzm**	Enterobacteria phage T4	64.9	37.0				1.70	164		[19]
**1lz1**	Homo sapiens	64.9	37.0				1.50	130		[20]
**4lyz**	Gallus gallus	74.8	41.0				2.00	129		[21]
1dzb	Meleagris gallopavo		41.0	68.3	77.3	74.8	2.00	129		[22]
1hhl	Numida meleagris		40.0	67.7	73.7	74.8	1.90	129		[22]
2gv0	Pelodiscus sinensis		31.5	62.4	43.2	74.8	1.90	131	x	[23]

Table S 2: Proteins of the Lysozyme family.

Myoglobin

PDB code	Host Organism	T_m^{exp} (°C)	T_{env} (°C)	$T_m^{(1)\,\text{est}}$ (°C)	$T_m^{(2)\,\text{est}}$ (°C)	$T_m^{(3)\,\text{est}}$ (°C)	Resol. ($\mathring{A}$)	Number of residues	Ligand [1]	Ref.
**2fal**	Aplysia Limacina	52.0					1.80	147	x	[24]
1uc3	Lampetra Fluvialis		9.5	48.8	42.3	52.0	2.30	149	x	[25]
3lhb	Petromyzon Marinus		15.5	52.2	50.0	52.0	2.70	149	x	[26]
2dc3	Human		37.0	65.8	80.3	82.2	1.68	193	x	
1ebt	Lucina Pectinata		26.6	59.4	66.0	52.0	1.90	142	x	[27]
3a5g	Tokunagayusurika Akamusi		22.5	56.8	60.3	82.2	1.81	152	x	[28]
2gnv	Oryza Sativa		29.0	60.9	69.3	52.0	2.30	165	x	[29]
1it2	Eptatretus Burgeri		21.0	55.9	58.2	52.0	1.60	146	x	[30]
1a6m	Physeter Catodon		35.0	64.6	77.6	82.2	1.00	151	x	[31]
2r4y	Scapharca Inaquivalvis		19.0	54.7	55.5	52.0	2.00	146	x	[32]
1myt	Thunnus Albacares		22.5	56.9	60.3	78.3	1.74	146	x	[33]
2nrl	Thunnus Atlanticus		22.5	56.9	60.3	78.3	0.91	147	x	[33]
**1ymb**	Horse Heart	78.3					1.90	153	x	[34]
1mdn	Sus Scrofa		37.0	67.1	83.2	78.3	1.98	153	x	[3]
1emy	Elephas Maximus		36.3	65.4	79.4	78.3	1.78	153	x	[35][36]
1mbs	Phoca Vitulina		37.8	66.3	81.5	78.3	2.50	153	x	[37]
1lhs	Caretta Caretta		23.0	57.2	61.0	78.3	2.00	153	x	[38]
2r80	Columba Livia		41.5	68.6	86.6	82.2	1.44	141	x	[39]
2zfb	Psittacula Krameri		40.0	67.7	84.5	78.3	3.00	141	x	[40]
1a4f	Anser Indicus		41.3	68.5	86.3	78.3	2.00	141	x	[41]
3k8b	Meleagris Gallopavo		41.0	68.3	85.9	78.3	2.30	141	x	[22]
1hbr	Chicken		41.0	68.3	85.9	78.3	2.30	141	x	[42]
3eok	Anas Platyrhynchos		41.9	68.8	87.2	78.3	2.10	141	x	[41]
**1bvc**	Sperm Whale	82.2					1.50	153	x	[43]

Table S 3: Proteins of the Myoglobin family.

β-Lactamase

PDB code	Host Organism	$T_m^{\exp}$ (°C)	T_{env} (°C)	$T_m^{(1)\,\mathrm{est}}$ (°C)	$T_m^{(2)\,\mathrm{est}}$ (°C)	$T_m^{(3)\,\mathrm{est}}$ (°C)	Resol. ($\AA$)	Number of residues	Ligand [1]	Ref.
**1blc**	Staphylococcus Aureus	41.6	33.5				2.20	257	x	[44][5]
3lez	Oceanobacillus Iheyensis		30.0	61.5	45.0	65.0	1.25	250	x	[45]
3byd	Klebsiella Oxytoca		10.0	49.1	16.9	65.0	1.93	257	x	[5]
3bfe	Citrobacter Sedlakii		33.5	63.7	50.0	65.0	2.40	262	x	[46]
3p09	Francisella Tularensis		37.0	65.8	54.9	41.6	1.90	290	x	[47]
3cjm	Enterococcus Faecalis V583		35.0	64.6	52.1	54.6	1.50	282	x	[48]
**1ke4**	Escherichia Coli	54.6	37.0				1.72	358	x	[49][50]
1fr1	Citrobacter Freundii		37.0	65.8	54.9	54.6	2.00	361		[51]
1bls	Enterobacter Cloacae		23.5	57.5	35.9	54.6	2.30	361	x	[52]
2qz6	Pseudomonas Fluorescens		28.0	60.3	42.2	54.6	2.26	358		[9]
3ozh	Yersina Pestis		37.0	65.8	54.9	54.6	1.91	351		[5]
3i7j	Mycobacterium Bovis		37.0	65.8	54.9	65.0	2.20	280		[53]
**4blm**	Bacillus Licheniformis	65.0	42.5				2.00	265	x	[54][9]
2gdn	Mycobacterium Tubercolosis		37.0	65.8	54.9	65.0	1.72	267		[55]
1dy6	Serrata Marcenses		38.5	66.8	57.0	65.0	2.13	267		[9]
1bsg	Streptomyces Albus G		45.0	70.8	66.2	65.0	1.85	266	x	[9]
2cc1	Mycobacterium Fortuitum		37.0	65.8	54.9	65.0	2.13	262		[9]
1shv	Klebsella Pneumoniae		45.0	70.8	66.2	65.0	1.98	265	x	[9]

Table S 4: Proteins of the β-lactamase family.

α-Lactalbumin

PDB code	Host Organism	$T_m^{\exp}$ (°C)	T_{env} (°C)	$T_m^{(1)\,\mathrm{est}}$ (°C)	$T_m^{(2)\,\mathrm{est}}$ (°C)	$T_m^{(3)\,(\mathrm{est})}$ (°C)	Resol. ($\AA$)	Number of residues	Ligand [1]	Ref.
**1hml**	Homo Sapiens	39.5	37.0				1.70	257	x	[56]
1alc	Papio cynocephalus		39.0	67.1	71.1	39.5	1.70	123		[57]
1hfy	Capra hircus		39.0	67.1	71.1	70.8	2.30	123		[58]
1hfx	Cavia porcellus		38.3	66.7	61.0	39.5	1.90	123		[59]
**1hfz**	Bos taurus	56.2	38.0				2.30	124		[58][60]
**1hmk**	Capra hircus	70.8	39.0				2.00	124		[58][61]

Table S 5: Proteins of the α-Lactalbumin family.

Acylphosphatase

PDB code	Host Organism	$T_m^{\exp}$ (°C)	T_{env} (°C)	$T_m^{(1)\,\mathrm{est}}$ (°C)	$T_m^{(2)\,\mathrm{est}}$ (°C)	$T_m^{(3)\,\mathrm{est}}$ (°C)	Resol. ($\AA$)	Number of residues	Ligand [1]	Ref.
**2acy**	Bos taurus	53.8	38.0				1.80	98	x	[62][58]
2vh7	Homo sapiens		37.0	65.8	52.8	53.8	1.45	99		
1aps	Equus caballus		38.3	66.6	54.1	53.8		98		[58]
1urr	Drosophila melanogaster		23.0	57.2	39.4	53.8	1.50	102	x	[63]
2gv1	Escherichia coli		37.0	65.8	52.8	100.8		92		[50]
1v3z	Pyrococcus horikoshii		98.0	103.7	111.4	111.5	1.72	96		[64]
2fhm	Bacillus subtilis		37.0	65.8	52.8	100.8		91		[5]
**2bjd**	Sulfolobus solfataricus	100.8	87.0				1.27	101	x	[62][65]
**1w2i**	Pyrococcus horikoshii	111.5	98.0				1.50	91	x	[64][66]

Table S 6: Proteins of the Acylphosphatase family.

Adenylate Kinase

PDB code	Host Organism	T_m^{exp} (°C)	T_{env} (°C)	$T_m^{(1)\text{ est}}$ (°C)	$T_m^{(2)\text{ est}}$ (°C)	$T_m^{(3)\text{ est}}$ (°C)	Resol. (Å)	Number of residues	Ligand [1]	Ref.
**1p3j**	Bacillus subtilis	47.6	37.0				1.90	217	x	[67][5]
**3fb4**	Jeotgalibacillus marinus	47.6	18.5	54.4	43.3	47.6	2.00	216	x	[68]
3gmt	Burkholderia pseudomallei 1710b		23.0	57.2	46.4	51.8	2.10	230	x	[69]
3be4	Cryptosporidium parvum Iowa II		37.0	65.8	56.3	47.7	1.60	217	x	[70]
2rh5	Aquifex aeolicus		80.0	92.5	86.6	43.3	2.48	206		[71]
2c9y	Homo sapiens		37.0	65.8	56.3	47.7	2.10	242	x	
1zak	Zea mays		28.0	60.3	50.0	51.8	3.50	222	x	[72]
1p4s	Mycobacterium tuberculosis		37.0	65.8	56.3	43.3		181		[73]
2xb4	Desulfovibrio gigas		35.5	64.9	55.2	74.5	1.80	223	x	[74]
3cm0	Thermus thermophilus		63.5	85.4	78.5	51.8	1.80	186		[75]
3adk	Sus scrofa		37.0	67.1	57.7	43.3	2.10	195	x	[?]
**1s3g**	Sporosarcina globispora	43.3	15.0				2.25	217	x	[67][76]
**1aky**	Saccharomyces cerevisiae	47.7	27.5				1.63	220	x	[77] [78]
**1ank**	Escherichia coli	51.8	37.0				2.00	214	x	[79][50]
**1zip**	Geobacillus stearothermophilus	74.5	51.0				1.85	217	x	[81]

Table S 7: Proteins of the Adenylate Kinase family.

Cell 12A Endoglucanase

PDB code	Host Organism	T_m^{exp} (°C)	T_{env} (°C)	$T_m^{(1)\,est}$ (°C)	$T_m^{(2)\,est}$ (°C)	$T_m^{(3)\,est}$ (°C)	Resol. (Å)	Number of residues	Ligand [1]	Ref.
**1oa3**	Trichoderma citrinoviride	49.2	40.0				1.70	218	x	[82][83]
2bw8	Rhodothermus marinus		67.5	84.7	71.5	70.4	1.54	227	x	[84]
**1h8v**	Trichoderma reesei	54.5	34.5				1.90	218	x	[85] [86]
**1oa4**	Streptomyces sp. 11AG8	66.8	30.0				1.50	222		[82][87]
2nlr	Streptomyces lividans		29.0	60.9	56.5	66.8	1.20	234	x	[88]
**1olr**	Humicola grisea	68.7	50.0				1.20	224		[85][89]
**1cec**	Clostridium thermocellum	70.4	60.0				2.15	343		[90][91]

Table S 8: Proteins of the Cell 12A Endoglucanase family.

Cold-Shock Protein

PDB code	Host Organism	T_m^{exp} (°C)	T_{env} (°C)	$T_m^{(1)\,est}$ (°C)	$T_m^{(2)\,est}$ (°C)	$T_m^{(3)\,est}$ (°C)	Resol. (Å)	Number of residues	Ligand [1]	Ref.
**1csp**	Bacillus subtilis	53.8	37.0				2.45	67		[92][5]
3l2z	Mycobacterium tuberculosis H37Rv		37.0	65.8	55.2	56.7	1.10	266		[93]
3a0j	Thermus thermophilus HB8		68.5	85.4	75.9	76.9	1.65	72		[75] [86]
1g6p	Thermotoga maritima		80.0	92.5	83.5	76.9		66		[94]
**1mjc**	Escherichia coli	56.7	37.0				2.00	69		[50][95]
**1c90**	Bacillus caldolyticus	76.9	70.0				1.17	66	x	[96][97][98]
3cam	Neisseria meningitidis MC58		37.0	65.8	55.2	56.7	2.60	67		[99]

Table S 9: Proteins of the Cold Shock Protein family.

Cytochrome P450

PDB code	Host Organism	T_m^{exp} (°C)	T_{env} (°C)	$T_m^{(1)\,est}$ (°C)	$T_m^{(2)\,est}$ (°C)	$T_m^{(3)\,est}$ (°C)	Resol. (Å)	Number of residues	Ligamd [1]	Ref.
**1bu7**	Bacillus megaterium	47.0	30.0				1.65	455	x	[100][101]
2q9g	Homo sapiens		37.0	65.8	59.7	88.0	2.40	456	x	
1ea1	Mycobacterium tuberculosis		37.5	66.1	60.0	55.0	2.21	455	x	[55]
1wiy	Thermus thermophilus HB8		68.5	85.4	85.2	88.0	2.00	389	x	[75]
2ve3	Synechocystis sp. PCC 6803		33.0	63.4	56.4	91.0	2.10	444	x	[102]
2bvj	Streptomyces venezuelae		32.0	62.7	55.6	55.0	2.10	436	x	[103]
1po5	Oryctolagus cuniculus		39.0	66.9	61.0	61.0	1.60	476	x	[104]
2rfb	Picrophilus torridus		60.0	80.1	78.3	91.0	2.50	343	x	[105]
2wv2	Trypanosoma brucei		27.0	59.6	51.6	61.0	2.70	475	x	[106]
3abb	Streptomyces avermitilis		37.0	65.8	59.7	55.0	2.30	408	x	[107]
1lfk	Amycolatopsis orientalis		28.0	60.3	52.4	55.0	1.70	398	x	[109]
1odo	Streptomyces coelicolor A3(2)		25.0	58.4	50.0	55.0	1.85	408	x	[111]
1pkf	Sorangium cellulosum		20.0	55.3	45.9	55.0	2.10	419	x	[5]
2z3t	Streptomyces sp. TP-A0274		40.0	67.7	62.1	55.0	1.90	425	x	[112]
2jjn	Saccharopolyspora erythraea		31.0	62.2	54.8	55.0	1.59	411	x	[5]
**1oxa**	Saccharopolyspora erythraea		55.0	31.0			2.10	403	x	[5][101]
3a4g	Pseudonocardia autotrophica		23.5	57.4	48.7	55.0	1.75	441	x	[5]
2zbx	Streptomyces griseolus		25.0	58.4	50.0	55.0	1.50	412	x	[5]
2x9p	Streptomyces natalensis		30.0	61.5	54.5	55.0	2.10	404	x	[5]
1jfb	Fusarium oxysporum		26.0	59.0	50.8	55.0	1.00	404	x	[113]
2fr7	Rhodopseudomonas palustris CGA009		33.5	63.7	56.8	55.0	2.01	412	x	[5]
1cpt	Pseudomonas sp.		37.0	65.8	59.7	47.0	1.80	428	x	[5]
**1akd**	Pseudomonas putida	61.0	30.0				1.80	414	x	[50][108]
3bdz	Citrobacter braakii		33.5	63.7	56.9	91.0	1.80	397	x	[114]
**1n97**	Thermus thermophilus HB27	88.0	68.5				2.60	389	x	[50][75]
1ue8	Sulfolobus tokodaii		80.0	92.5	94.5	91.0	3.00	367	x	[115]
**1f4t**	Sulfolobus solfataricus	91.0	77.5				1.93	368	x	[50][65]
2wiv	Rhodococcus		31.0	62.1	54.8	55.0	1.90	394	x	[5]
3o1a	Actinoplanes teichomyceticus		30.0	61.5	54.0	55.0	2.50	417	x	[5]

Table S 10: Proteins of the Cytochrome P450 family.

Ribonuclease

PDB code	Host Organism	T_m^{exp} (°C)	T_{env} (°C)	$T_m^{(1)\,\mathrm{est}}$ (°C)	$T_m^{(2)\,\mathrm{est}}$ (°C)	$T_m^{(3)\,\mathrm{est}}$ (°C)	Resol. ($\AA$)	Number of residues	Ligand [1]	Ref.
**1rgg**	Streptomyces aureofaciens	48.4					1.20	96	x	[116]
**9rnt**	Aspergillus oryzae	51.6					1.50	104		[117]
1rds	Aspergillus phoenicis		33.5	63.7	52.5	51.6	1.80	105	x	[118]
1fus	Fusarium fujikuroi		30.0	61.5	50.0	102.0	1.30	106		[119]
**1rnh**	Escherichia Coli	53.2					2.00	155	x	[121]
**1rbn**	Bos taurus	62.8					2.10	124	x	[120]
3h08	Chlorobaculum tepidum		47.5	72.3	62.0	53.2	1.60	146		[122]
1hrh	Human immunodeficiency virus 1		37.0	65.9	54.8	62.8	2.10	136		
2hb5	Moloney murine leukemia virus		37.0	65.9	54.8	62.8	1.59	164	x	
**2ehg**	Sulfolobus tokodaii str. 7	102.0					1.60	149		[123]

Table S 11: Proteins of the Ribonuclease family.

**Protein**	$T_m^{\exp}$ (^oC)	$T_m^{\Delta\nabla}$ (^oC)	$T_m^{\Diamond}$ (^oC)	$T_m^{\Delta\nabla}$ (^oC)
PDB code		jack knife	jack knife	no jack knife
1aqh	43.7	53.2	60.3	50.3
1ppi	65.6	55.8	58.7	60.8
1jae	65.9	71.2	64.0	67.1
1smd	70.3	67.8	64.1	71.6
1am7	52.3	53.5	69.0	47.8
2lzm	64.8	89.6	64.1	68.2
1lz1	64.9	57.7	62.5	58.8
4lyz	74.8	61.8	54.5	73.7
2fal	52.0	66.2	80.5	52.2
1ymb	78.3	58.3	61.4	65.8
1bvc	82.2	106.5	76.3	87.3
1blc	41.6	50.3	79.5	45.3
1ke4	54.6	48.8	56.0	51.1
4blm	65.0	59.9	51.9	63.7
1bmc	51.0	50.4	54.3	50.5
1hml	39.5	51.1	65.8	39.6
1hfz	56.2	76.7	56.1	63.9
1hmk	70.8	51.2	45.6	61.5
2acy	53.8	61.8	91.9	54.9
2bjd	100.8	93.3	85.5	104.4
1w2i	111.5	118.3	96.7	108.0
1p3j	47.6	54.4	53.6	54.9
3fb4	47.6	54.5	49.6	53.7
1s3g	43.4	53.7	52.7	54.1
1aky	47.7	51.6	52.2	54.4
1ank	51.8	49.9	28.1	54.5
1zip	74.8	48.7	48.6	57.8
1oa3	49.2	52.4	63.8	46.0
1h8v	54.5	57.5	50.1	50.9
1oa4	66.8	90.3	59.0	59.8
1olr	68.7	54.1	52.1	65.8
1cec	70.4	57.6	69.8	74.3
1csp	53.8	60.3	59.2	58.0
1mjc	56.7	64.6	61.7	60.7
1c9o	76.9	54.0	11.3	73.8
1bu7	47.0	70.9	58.9	56.9
1oxa	55.1	72.4	66.8	69.8
1akd	61.0	53.8	53.6	59.6
1n97	88.5	61.9	55.8	89.8
1f4t	91.2	79.6	64.0	69.2
1rgg	48.4	47.8	64.1	51.1
9rnt	51.6	47.6	80.0	53.2
1rnh	53.2	55.8	32.7	53.7
1rbn	62.8	50.0	54.5	57.6
2ehg	102.0	138.2	51.9	102.9

Table S 12: Experimental and predicted T_m's of the proteins belonging to the 11 homologous families. In the first column the experimental T_m values are reported; in the second, third and last columns the T_m's computed from the $\Delta G^{\Delta\nabla}$ (in cross-validation), $\Delta G^{\Diamond}$ (in cross-validation) and $\Delta G^{\Delta\nabla}$ (in direct-validation) are listed.

Family	$\bar{T}_m(S_f^{\triangle})$	$\bar{T}_m(S_f^{\diamond})$	$\bar{T}_m(S_f^{\nabla})$
01) Acylphosphatase	51.5	65.3	79.3
02) Ribonuclease	52.2	66.1	80.0
03) Lysozyme	51.6	65.5	79.5
04) Endoglucanase	52.0	65.8	79.4
05) Adelinate Kinase	51.8	65.6	79.3
06) α-amylase	52.1	64.8	79.9
07) α-lactalbumina	51.9	65.3	79.3
08) Myoglobine	52.2	65.4	79.8
09) CytoChrome	51.1	65.7	77.9
10) β-lactamase	48.6	64.1	77.6
11) Cold Schock	52.1	65.7	79.6
**Sum**	**51.6**	**65.4**	**79.2**

Table S 13: Average melting temperature $\bar{T}_m$ of the proteins belonging to the different datasets S_f.

**Family**	$T_m^{(2)\,\mathrm{est}}$	N
α-Amylase	1.47 T_{env} + 14.2 °C	4
Lysozyme	3.53 T_{env} - 69.9°C	4
Myoglobin	1.38 T_{env} + 29.2 °C	3
β-Lactamase	1.41 T_{env} + 2.74 °C	4
α-Lactalbumin	15.6 T_{env} - 538.8 °C	3
Acylphosphatase	0.96 T_{env} + 17.2 °C	3
Adenylate kinase	0.70 T_{env} + 30.2 °C	6
Cell 12A endoglucanase	0.39 T_{env} + 45.2 °C	5
Cold shock protein	0.66 T_{env} + 31.0 °C	3
Cytochrome P450	0.86 T_{env} + 28.2 °C	5
Ribonuclease	1.03 T_{env} + 24.6 °C	5

Table S 14: Family-dependent T_m-T_{env} regression lines that define the estimated melting temperature $T_m^{(2)\,\mathrm{est}}$ as a function of T_{env}. N is the number of proteins in the family.

References

[1] Laskowski RA (2001), PDBsum: summaries and analyses of PDB structures, Nucleic Acids Res. 29, 221222.

[2] S. D'Amico, C. Gerday and G. Feller, The Journal of Biological Chemistry, 276, 25791-25796 (2001).

[3] http://www.oxfordsandyblackpigs.org.uk/pig_body_temperature.htm

[4] S. D'Amico, C. Gerday and G. Feller, Biochemistry. 38(14), 4613-9 (1999).

[5] J.G. Holt, H.R. Kreyg, , R.H.A. Sneath, J.T. Staley, and S.T. Williams, Bergey's Manual of Determinative Bacteriology (1994).

[6] R.H. Sajedi, H. Naderi-Manesh, K. Khajeh, R. Ahmadvand, B. Ranjbar, A. Asoodeh, F. Moradian, Enzyme and Microbial Technology 36, 666671 (2005).

[7] P. Barbesgaard, H.P. Heldt-Hansen, and B. Diderichsen, Appl. Microbiol. Biotechnol. 36, 569-572, (1992).

[8] M. Blanc, L. Marilley, T. Beffa, M. Aragno, Int. J. Syst. Bacteriol. 47(4), 1246-8 (1997).

[9] G. Vogt, S. Woell, P. Argos, J. Mol. Biol. 269(4), 631-43 (1997).

[10] K. Am, K. Saeki, K. Igarashi, M. Takaiwa, T. Uemura, H. Hagihara, S. Kawai, S. Ito, Biochimica et Biophysics Acta, 1243, 315-324 (1995).

[11] genome.jgi-psf.org/halor/halor.home.html

[12] A. Astoreca, C. Magnoli, M.L.. Ramirez, M. Combina, A. Dalcero, International Journal of Food Microbiology 119, 314318 (2007).

[13] http://www.ogtr.gov.au/internet/ogtr/publishing.nsf/content/barley-3/$FILE/biologybarley08.pdf

[14] K. Saeki , J. Hitomi, M. Okuda, Y. Hatada, Y. Kageyama, M. Takaiwa, H. Kubota, H. Hagihara, T. Kobayashi, S. Kawai, S. Ito, Extremophiles, 6(1), 65-72 (2002).

[15] W. Zillig,I. Holz, H.P. Klenk, J. Trent, S. Wunderl, D. Janekovic, E. Imsel, B. Haas, Syst. Appl. Microbiol. 9, 62 (1987).

[16] S. Balamurugan, M.E.. Dugan, J. Basic Microbiol. 50(6), 507-18 (2010).

[17] C. Evrard, J. Fastrez, P. Soumillion, FEBS Lett. 460(3) (1999).

[18] hypertextbook.com/facts/1998/PeiJunChen.shtml

[19] M. Matsumura, W.J. Becktel, B.W. Matthews, Nature 334(6181), 406-10 (1988).

[20] K. Takano, K. Ogasahara, H. Kaneda, Y. Yamagata, S. Fujii, E. Kanaya, M. Kikuchi, M. Oobatake, K. Yutani, J. Mol. Biol. 254(1), 62-76 (1995).

[21] A. Rajpal, J.F. Kirsch, Proteins: Structure, Function, and Bioinformatics, 40(1), 4957 (2000).

[22] M.W. Dietz, M. Van Kampen, J. Comp. Physiol. B 164, 69-75 (1994).

[23] C.H. Ernst, J.E. Lovich, Turtles of the United States and Canada,

[24] R.A. Staniforth, S. Giannini, M.G. BigottiG, F.. Cutruzzola, C. Travaglini-Allocatelli, M. Brunori, J. Mol. Biol. 297(5), 1231-44 (2000).

[25] P.N. Claridge, I.C. Potter, J. exp. Biol. 63, 193-206 (1975).

[26] G.J. Farmer, F.W.H. Beamish, P.F. Lett, Journal of the Fisheries Research Board of Canada 34, 1373-1378 (1977).

[27] S.F. Rondinelli, F. Barros, Journal of Sea Research 64, 401407 (2010).

[28] P.D. Armitage, P.S. Cranston, L.C.V. Pinder, The Chironomidae: biology and ecology of non-biting midges

[29] www.globinmed.com/index.php?option=com_content&view=article&id=79911:oryza-sativa-l&Itemid=116

[30] B. Fernholm, Marine Biology 27, 351-356 (1974).

[31] C.G. Flewellen and R.J. Morris, Deep-Sea Research 25, 269-277 (1978).

[32] B. Vismann, Mar. Ecol. Prog. Ser. 98, 115-122 (1993).

[33] B.A. Block, J.E. Keen, B. Castillo, H. Dewar, E.V. Freund, D.J. Marcinek, R.W. Brill, C. Farwell, Marine Biology 130, 119-132 (1997).

[34] J.W. Tanner, J.S. Johansson, P.A. Liebman, R.G. Eckenhoff, Biochemistry. 40(16), 5075-80 (2001).

[35] P.A. Rees, Journal of Thermal Biology 27, 353358 (2002).

[36] W.H. Elder, D.H. Rodgers, Mammalia 39, 395399 (1975).

[37] K. Miller, M. Rosenmann, P. Morrison, Comparative Biochemistry and Physiology Part A: Physiology 54 (1), 105-107 (1976).

[38] K. Sato, W. Sakamoto, Y. Matsuzawa, H. Tanaka, S. Minamikawa, Y. Naito, Marine Biology 123 (2) 197-205 (1995).

[39] M.E. Rashotte, I.F. Pastukhov, E.L. Poliakov, R.P. Henderson, J. Physiol. Regul. Integr. Comp. Physiol. 275, R1690-R1702 (1998).

[40] W.W. Weathers, D.F. Caccamise, Oecologia 35 (2), 173-183 (1978).

[41] G.R. Scott, V. Cadena, G.J. Tattersall, W.K. Milsom, The Journal of Experimental Biology 211, 1326-1335 (2008).

[42] chickscope.beckman.uiuc.edu/explore/embryology/day02/comparative.html

[43] R.J. Pinker, L. Lin, G.D. Rose, N.R. Kallenbach, Protein Sci. 2(7), 1099-105 (1993).

[44] J. Rahil, R.F. Pratt, Biochemistry, 33(1), 116-25 (1994).

[45] J. Lu, Y. Nogi, H. Takami, Microbiology Letters 205, 291-297 (2001).

[46] M. Dworkin, S. Falkow, The prokaryotes: a handbook on the biology of bacteria, Springer-Verlag (1992).

[47] M.P. Payne, R.J. Morton, J. Vet. Diagn. Invest. 4, 264-269 (1992).

[48] K.H. Schleifer, R. Kilpper-Balz, Int. J. of Systematic Bacteriology, 31-34 (1984).

[49] B.M. Beadle, S.L. McGovern, A. Patera, B.K. Shoichet, Protein Sci. 8(9), 1816-24 (1999).

[50] M. Ferrer, T.N. Chernikova, M.M. Yakimov, P.N. Golyshin, K.N. Timmis, Nature Biotechnology 21, 1266 - 1267 (2003).

[51] C.T. Verrips, R.H. Kwast, W. Vries, Antonie van Leeuwenhoek 46 (6), 551-563 (1980).

[52] A. Bevilacqua, M. Cannarsi, M. Gallo, M. Sinigaglia, A.R. Corbo, Journal of Food Science 75 (1) (2010).

[53] W.A. Hagan, D.W. Bruner, J.F. Timoney, Hagan and Bruner's microbiology and infectious diseases of domestic animals, Cornell University Press (1981).

[54] L.M. Ellerby, W.A. Escobar, A.L. Fink, C. Mitchinson, J.A. Wells, Biochemistry, 29(24), 5797-806 (1990).

[55] M.G. Sonnenberg, J.T. Belisle, Infect Immun. 65(11), 4515-24 (1997).

[56] Y. Harushima, S. Sugai, Biochemistry, 28(21), 8568-76 (1989).

[57] P.G. Hiley, The Journal of Physiology, 254, 657-671 (1976).

[58] www.circadian.org/animal.html

[59] J.E. Harkness, J.E. Wagner, The Biology and Medicine of Rabbits and Rodents, Wiley-Blackwell Ed. (2010).

[60] L.H. Greene, J.A. Grobler, V.A. Malinovskii, J. Tian, K.R. Acharya, K. Brew, Protein Eng. 12(7), 581-7 (1999).

[61] A. Vanhooren, A. Chedad, V. Farkas, Z. Majer, M. Joniau, H. Van Dael, I. Hanssens, Proteins, 60(1), 118-30 (2005).

[62] A. Corazza *et all*, Proteins, 62(1), 64-79 (2006).

[63] J.D. Kelty, E.L. Richard, Journal of Insect Physiology 45, 719726 (1999).

[64] J.M. Gonzlez *et all*, Extremophiles 2, 123130 (1998).

[65] I. Udaa *et all*, Lipids, 36 (1) (2001).

[66] Y.Y. Cheung , S.Y. Lam , W.K. Chu , M.D. Allen, M. Bycroft , K.B. Wong, Biochemistry, 44 (12), 46014611 (2005).

[67] E. Bae, G.N. Phillips Jr., J. Biol. Chem. 279, 28202-28208 (2004).

[68] J.H. Yoon *et all*, International Journal of Systematic and Evolutionary Microbiology 51, 20872093 (2001).

[69] L.D. Sprague, H. Neubauer, J. Vet. Med. B Infect. Dis. Vet. Public Health. 51(7), 305-20 (2004).

[70] M.S. Abrahamsen *et all*, Science, 304 (2004).

[71] G. Peng *et all*, Biochemistry 42, 3032-3039 (2003).

[72] A.K. Hardacre, H.L. Turnbull, Annals of Botany 58 (6), 779-787 (1986).

[73] www.textbookofbacteriology.net/nutgro5.html

[74] A. Mukhopadhyay *et all*, J. Biol. Inorg. Chem. 16, 5161 (2011).

[75] T. Oshima, K. Imahori, International Journal of Systematic Bacteriology, 24 (1), 102-112, (1974).

[76] www.tgw1916.net/Bacillus/globisporus.html

[77] P. Spuergin, U. Abele, G.E. Schulz, Eur. J. Biochem. 231(2), 405-13 (1995).

[78] S.L. Tai, P. Daran-Lapujade, M.C. Walsh, J.T. Pronk, J.M. Daran, Molecular Biology of the Cell 18, 51005112 (2007).

[79] T. Rose, P. Glaser, W.K. Surewicz, H.H. Mantsch, J. Reinstein, K. Le Blay, A.M. Gilles, O. Barzu, J. Biol. Chem. 266(35), 23654-9 (1991).

[80] M. Blanc, L. Marilley, T. Beffa, M. Aragno, Int. J. Syst. Bacteriol. 47(4), 1246-8 (1997).

[81] P. Glaser , E. Presecan , M. Delepierre , W.K. Surewicz , H.H. Mantsch , O. Barzu , A.M. Gilles, Biochemestry 31, 3038-3043 (1992).

[82] M. Sandgren *et all*, Protein Sci. 12(4), 848-60 (2003).

[83] R.M. Danielson, C.B. Davey, Soil Biol. Biochern. 5 (5), 495-504 (1973).

[84] Z. Silva *et all*, Extremophiles 3, 163172 (1999).

[85] M. Sandgren *et all*, Protein Sci. 12(12), 2782-93 (2003).

[86] J.P. Smits *et all*, Enzyme and Microbial Technology 22, 50-57 (1998).

[87] M. Sandgren, Structural and Functional Studies of Glycoside Hydrolase Family 12 Enzymes from Trichoderma reesei and other Cellulolytic Microorganisms, Acta Univetsitatis Uppsaliensis, Uppsala (2003).

[88] D. Kluepfel, F. Shareck, F. Mondou, R. Morosoli, Appl. Microbiol. Biotechnol. 24, 230-234 (1986).

[89] R. Da Silva, D.K. Yim, Y.K. Park, J. of Fermentation and Bioengineering, 77 (1), 109-111 (1994).

[90] A. Nemeth, S. Kamondi, A. Szilagyi, C. Magyar, Z. Kovari, P. Zavodszky, Biophys. Chem. 132, 229-241 (2002).

[91] D.B. Levina, R. Islamc, N. Cicekc , R. Sparling, International Journal of Hydrogen Energy 31, 14961503 (2006).

[92] M. Wunderlich, A. Martin, F.X. Schmid, J. Mol. Biol. 347(5), 1063-76 (2005).

[93] S. Balamurugan, M.E. Dugan, J. Basic Microbiol. 50(6), 507-18 (2010).

[94] R. Paul, D. Lazarev, S. Altman, Nucleic Acids Research 29 (4), 880-885 (2001).

[95] D.M. Vu, K.L. Reid, H.M. Rodriguez, L.M. Gregoret, Protein Sci. 10(10), 2028-36 (2001).

[96] B.N. Dominy, D. Perl, F.X. Schmid, C.L. Brooks, J. Mol Biol. 319(2), 541-54 (2002).

[97] M. Wunderlich, F.X. Schmid, Protein Eng. Des. Sel. 19(8), 355-8 (2006).

[98] A. Weerkamp, W. Heinen, J. Bacteriology, 109(1), 443-446 (1972).

[99] M. Guckenberger *et all*, J. Bacteriology 184(9), 25462551 (2002).

[100] www.tgw1916.net/Bacillus/megaterium.html

[101] J.K. Yano *et all*, The Journal of Biological Chemistry, 278, 608-616 (2003).

[102] M. Lopo *et all*, J. Mol. Microbiol. Biotechnol. 22(2), 71-82 (2012).

[103] A. MacIntosh, A study on the growth and metabolic activity of Streptomyces Venezuelae, Dalhousie University (2010).

[104] http://wildpro.twycrosszoo.org/S/0MLagomorph/Leporidae/Oryctolagus/Oryctolagus_cuniculus/Oryctolagus_

[105] E. Serour, G. Antranikian, Antonie Van Leeuwenhoek, 81(1-4), 73-83 (2002).

[106] G.A.M. Crossa, J.C. Manninga, Parasitology, 67 (3), 315-331 (1973).

[107] R.W. Burg, Antimicrob Agents Chemother. 15(3), 361367 (1979).

[108] P. Fonseca, R. Moreno, F. Rojo, Environmental Microbiology Reports 3 (3), 329339 (2011).

[109] web.mst.edu/

[110] M. Zaparty *et all*, Extremophiles, 14(1), 119142 (2010).

[111] bacmap.wishartlab.com/organisms/106

[112] T. Satyanarayana, B.N. Johri, Microbial Diversity, IPH (2005).

[113] www.agroatlas.ru/en/content/diseases/Fabacee/Fabacee_pisi_Fusarium_spp/

[114] S. Falkow, E. Rosenberg, K.H. Schleifer, E. Stackebrandt, M. Dworkin, The Prokaryotes, Springer (2006).

[115] T. Suzuki *et all*, Extremophiles, 6(1), 39-44 (2002).

[116] E.J. Hebert, A. Giletto, J. Sevcik, L. Urbanikova, K.S. Wilson, Z. Dauter, C.N. Pace, Biochemestry, 37, 16192-16200 (1998)

[117] Y. Yu, G.I. Makhatadze, C.N. Pace, P.L. Privalov, Biochemistry. 33(11), 3312-9 (1994).

[118] A.C. Rizzatti, V.C. Sandrim, J.A. Jorge, H.F. Terenzi, M.L. Polizeli, J. Ind. Microbiol. Biotechnol. 31(2), 88-93 (2004)

[119] V.K. Gupta, A.K. Misra, R.K. Gaur, Journal of Plant Protection research, 50, 4 (2010)

[120] G. Gotte, M. Donadelli, D.V. Laurents, F. Vottariello, M. Morbio, M. Libonati, Biochemistry, 45(36), 10795-806 (2006).

[121] M. Haruki *et all*, European Journal of Biochemistry, 220(2), 623631 (1994).

[122] microbewiki.kenyon.edu/index.php/Chlorobium_tepidum.

[123] A. Mukaiyama, M. Haruki, M. Ota, Y. Koga, K. Takano, S. Kanaya, Biochemistry, 45(42), 1267312679 (2006)
